# Synergy of the flow behaviour and disperse phase of cellulose nanoparticles in enhancing oil recovery at reservoir condition

**DOI:** 10.1371/journal.pone.0220778

**Published:** 2019-09-27

**Authors:** Augustine Agi, Radzuan Junin, Agus Arsad, Azza Abbas, Afeez Gbadamosi, Nur Bashirah Azli, Jeffrey Oseh

**Affiliations:** 1 Department of Petroleum Engineering, School of Chemical and Energy Engineering, Faculty of Engineering, Universiti Teknologi Malaysia, Johor Bahru, Malaysia; 2 Institute for Oil and Gas, Universiti Teknologi Malaysia, Johor Bahru, Malaysia; 3 Sudan University of Science and Technology, Khartoum, Sudan; Pandit Deendayal Petroleum University, INDIA

## Abstract

Ascorbic acid was used for the first time to synthesize cellulose nanoparticles (CNP) extracted from okra mucilage. The physical properties of the CNP including their size distribution, and crystalline structures were investigated. The rheological properties of the cellulose nanofluid (CNF) were compared with the bulk okra mucilage and commercial polymer xanthan. The interfacial properties of the CNF at the interface of oil-water (O/W) system were investigated at different concentrations and temperatures. The effects of the interaction between the electrolyte and ultrasonic were determined. Core flooding experiment was conducted at reservoir condition to justify the effect of the flow behaviour and disperse phase behaviour of CNF on additional oil recovery. The performance of the CNF was compared to conventional EOR chemical. The combined method of ultrasonic, weak-acid hydrolysis and nanoprecipitation were effective in producing spherical and polygonal nanoparticles with a mean diameter of 100 nm, increased yield of 51% and preserved crystallinity respectively. The zeta potential result shows that the CNF was stable, and the surface charge signifies long term stability of the fluid when injected into oil field reservoirs. The CNF, okra and xanthan exhibited shear-thinning and pseudoplastic behaviour. The IFT decreased with increase in concentration of CNF, electrolyte and temperature. The pressure drop data confirmed the stability of CNF at 120°C and the formation of oil bank was enough to increase the oil recovery by 20%. CNF was found to be very effective in mobilizing residual oil at high-temperature high-pressure (HTHP) reservoir condition. The energy and cost estimations have shown that investing in ultrasonic-assisted weak-acid hydrolysis is easier, cost-effective, and can reduce energy consumption making the method economically advantageous compared to conventional methods.

## Introduction

Although there is a clamour for renewable energy, the dawn of this century has seen an increasing demand for fossil fuel. But the production from most oil fields around the world is declining because of high capillary forces and heterogeneity of the reservoirs leading to early abandonment [1, 2]. The challenge is to recover trapped oil economically and delay the abandonment. Hitherto, polymer and surfactant have been used to mobilize trapped oil by reducing the mobility ratio and the force at the interface of oil and water (O/W) respectively. But, the loss of surfactant by retention, sorption in the reservoir, the susceptibility of polymer to salinity and temperature. The high cost of these surfactants and polymers have made their use less economical due to the dwindling oil price [1].

Nanotechnology is one of the most innovative technologies of this decade, as the tailoring of chemicals and formulation of chemical slugs is seen as a lasting solution to the numerous problems plaguing the oil and gas industry [1, 3]. This is because nanoparticles can localize at O/W interfaces and reduce residual oil saturation. Experimental results have shown that nanoparticles can reduce IFT by 33–42% compared to brine [1, 4]. Nanoparticles can increase the viscosity of brine and decrease the viscosity of crude oil emulsion. However, the high cost of these nanoparticles (inorganic, metal and metal oxides), coupled with environmental concern have limited full-scale field application in enhanced oil recovery (EOR). Researchers have turned to nanoparticles, derived from a natural source, as an alternative.

Cellulose nanoparticles (CNP) has generated attention from the industries and researchers. This is because of their ability to be produced from diverse starting raw materials. Their adoptive surface chemistry, sustainability, biodegradability, renewability biocompatibility, and nontoxicity has made them a better substitute to synthetic nanoparticles and CNP are more economical to most high-performance nanoparticles [5]. The extraction methods can lead to different properties as molecular details such as alignment, hydrophobicity, hydrophilicity, particle size and shape affect IFT and rheological behaviour. Therefore, the viscosity and IFT of cellulose nanofluid (CNF) is highly dependent on the extraction and modification protocol [6]. The main method adopted to produce CNP is acid hydrolysis [7, 8]. This approach can produce nanoparticles ranging in size from 5–7 nm, but the drawback is the long duration and low yield of the nanoparticles [9, 10]. Also, the use of chemicals is a source of concern as nanoparticles obtained by classic acids (hydrochloric and sulphuric acids) are limited for practical utilization, because the nanoparticles have a strong tendency to aggregate, especially in dry powder form [11]. Researchers have turned to the use of physical treatment such as ultrasonic. It is a very effective method for the physical disruption of cellular structures [12]. The exposure of natural polymers’ solution to high intensity ultrasonic can reduce the molar mass. The preparation time becomes shortened, and the ultrasonic can effectively prevent aggregation. The modification of the process parameters and homogenization can reduce the disruption of crystallinity by ultrasonic (Kim et al., 2013). However, Kim et al. [13] and Goncalves et al. [14] reported disruption of the nanoparticle’s crystallinity by ultrasonic. But, the modification and intensification of the process parameters and homogenization can also enhance crystallinity by ultrasonic [13, 14]. If the crystallinity of nanoparticles is preserved after treatment, the powder products could be readily obtained and their accessibility to industrial items such as composites, nano-fillers, emulsifiers, viscocifiers and stabilizers could be improved.

Nanofluid flooding has been proven to be very effective in EOR. The performance depends on the material and formulation process. Unlike the detailed studies on interfacial and rheological properties of inorganic, metal and metal oxide in EOR, the use of nanofluid from a natural source has not been thoroughly examined for possible application in the oil industry. Therefore, in this study ascorbic acid was used for the first time to synthesize CNP assisted with ultrasonic and nanoprecipitation. The size distribution and crystalline structure of the CNF were investigated. The isolation, recovery yield and the impact of the process variables were studied. The rheological behaviours of the CNF were compared with the bulk okra mucilage, and commercial polymer xanthan in order to determine the connection between CNP morphology and their rheological behaviours in solutions. Interfacial properties of the CNF at the interface of O/W system was investigated at different concentrations and temperatures. The efficiency of electrolyte and ultrasonic interactions were also determined. Core flooding experiment was conducted at reservoir condition to justify the effect of the flow behaviour and disperse phase behaviour of CNF on additional oil recovery. The performance of the CNF was compared to conventional EOR chemical. The Energy and cost estimation of the method was also determined and compared with a conventional approach.

## Materials and methods

### Materials

Okra (or ladies’ finger), pineapple fruits and oranges were purchased from the Monday Market Taman Teratai, Johor Bahru, Malaysia. The lemongrass was obtained from Universiti Teknologi Malaysia (UTM) Johor Bahru campus. Palm wine, with a purity of 94% was used in place of alcohol and surfactant. It was obtained from Kangkar Pulai, Johor. Xanthan gum was supplied by R & M Marketing, Essex, U.K. Sodium chloride (NaCl) was supplied by QREC (Asia) Sdn. Bhd., Selangor, Malaysia with a molecular weight of 58.44 g/mol and a purity of 99% assay. Vinegar with a molecular weight of 60.05 g/mol, acetic acid (5%) and density of 1.0446 g/cu @ 25°C was supplied by PubChem. A West Lutong crude oil sample with density of 0.8283g/mL @ 25°C, API gravity of 37.7 and viscosity of 10 cp @ 25°C was obtained from Sarawak oil field in Malaysia. Core samples from a sandstone formation located in Sarawak, Malaysia were used. Table 1 shows the properties of the core samples. Deionized water (DIW) was used for all the samples’ preparation whereas, distilled water (DW) was used to prepare the dynamic light scattering (DLS) samples. NaCl, xanthan, vinegar, crude oil, and palm wine were used without further purification.

**Table 1:** Properties of core samples.

Properties	Core #1	Core #2	Core #3
**Length (cm)**	9.7	9.8	9.9
**Diameter (cm)**	3.7	3.7	3.7
**Bulk Volume (cm**^**3**^**)**	104.30	105.37	106.45
**Pore Volume (cm**^**3**^**)**	16.03	16.02	16.01
**Porosity (%)**	15.3	15.2	15.0
**Permeability (mD)**	167.43	152.24	102.53
**Initial Oil Saturation (%)**	98.13	93.44	95.00
**Injection rate (mL/min)**	0.5	0.5	0.5

## Methods

### Formulation of okra natural polymer

**Fig 1:**
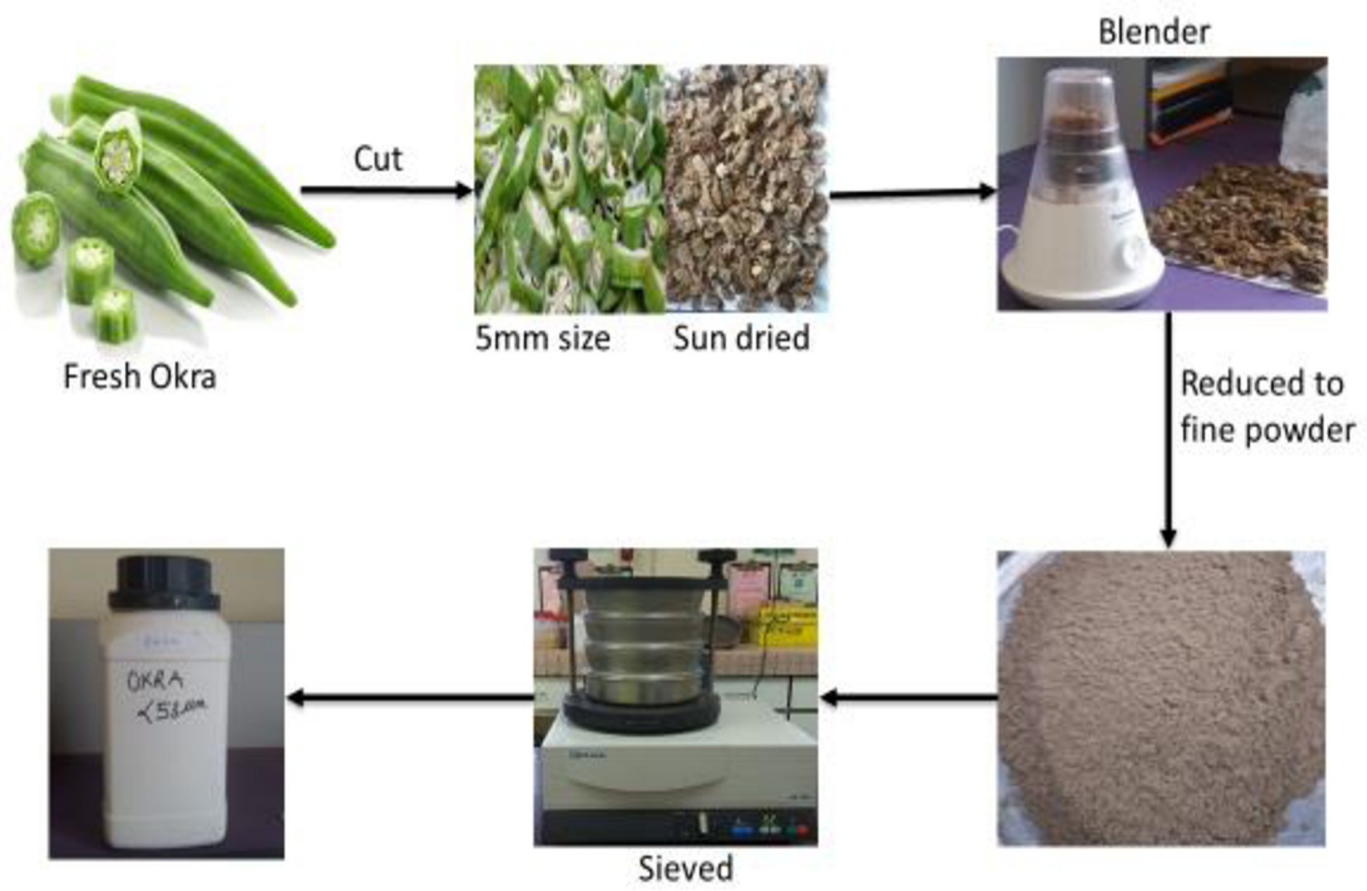
Okra sample preparation route.

The okra fruits were washed with DW and sliced into 5 mm thickness using a sterilized knife. The sliced vegetable fruit was placed on a flat plate sample holder and sun-dried to remove the moisture. The dried sample was ground into powder form using a blender. It was then passed through a 60 μm mesh sieve size (British Standard) to obtain a fine powder. The fine powder was stored in an airtight container. The illustration of the sample preparation route is shown in Fig 1.

### Extraction of ascorbic-acid from plant and fruit extract

The pineapples and oranges were washed with DW, and the skin removed by peeling with a sterilized knife to expose the flesh. The flesh was cut into chunks and blended to obtain the juice. The juice was collected and sieved to get a clear solution, poured into a container and refrigerated at 4°C. The lemongrass was hand-picked, and it was washed thoroughly with DW to remove dirt and impurities. A portion of 30 g of the washed lemongrass was finely cut and boiled in 100 ml of distilled water for 20 minutes. The extract was collected and sieved through a No.1 filter paper to remove particle matter and get a clear solution. The solution was refrigerated at 4°C for analysis.

### CNP production

**Fig 2:**
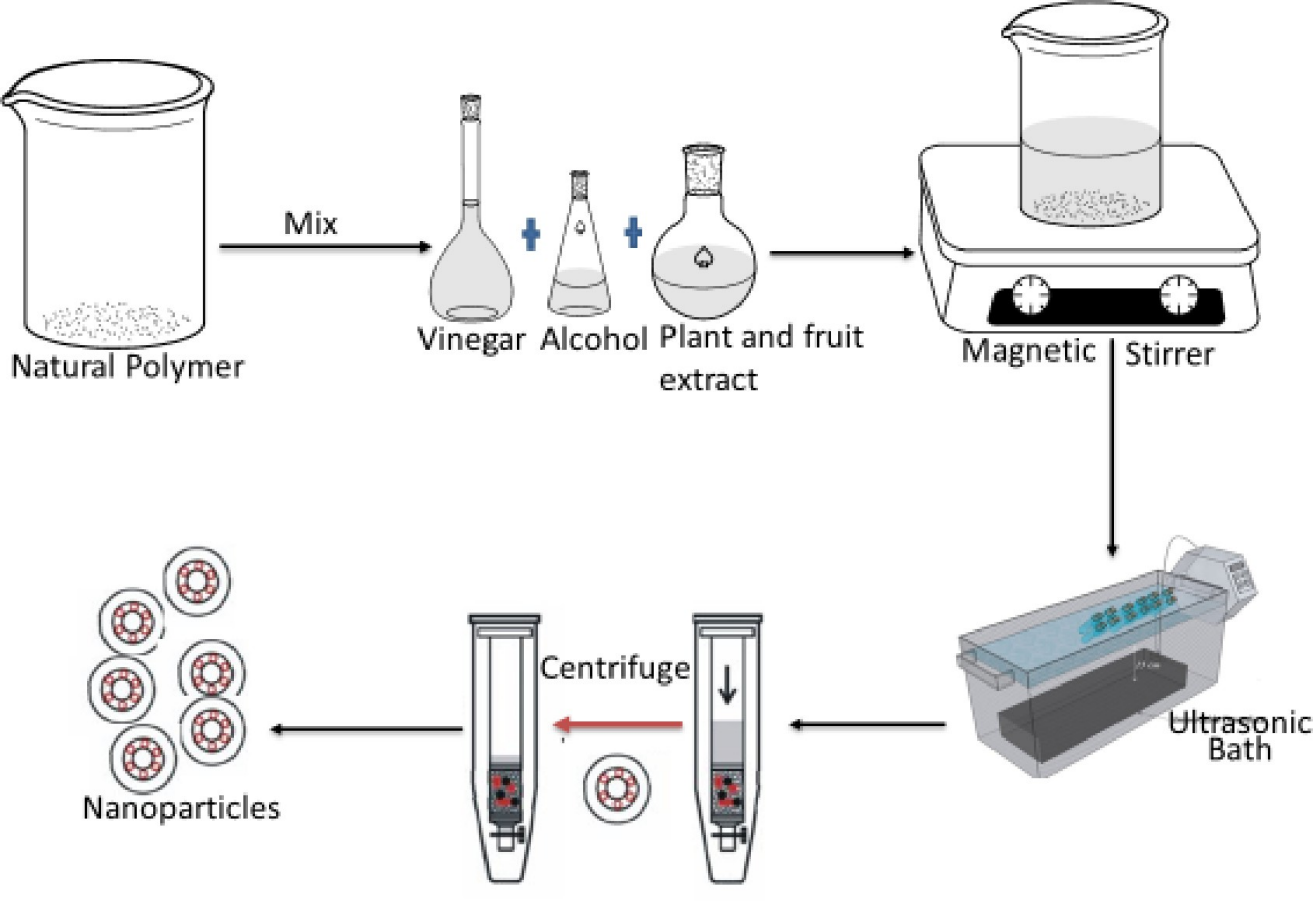
Synthesis schematic route for CSNP.

The technique used here was the combined method of nanoprecipitation and hydrolysis assisted ultrasonic using weak acid, alkaloids, and enzymes from plant and fruits extracts. The dried okra sample was soaked in 1 litre of distilled water for 20 minutes, filtered to produce mucilage. The mucilage was dissolved in 20 mL of vinegar to form a solution. The solution was added dropwise into a fixed quantity of absolute alcohol (palm wine). The plant extract was added slowly to the solution at a ratio of 1:10 (v/v) for bio-reduction. The mixture was stirred continuously using a magnetic stirrer at a constant stirring rate (1100 rpm), temperature of 60°C for 120 hours. The mixture was then placed in an ultrasonic bath (W: 21 cm x L: 50 cm x H: 30 cm). A Crest Genesis^TM^ XG-500-6 ultrasonic generator provided the ultrasound for three hours. The generator produces ultrasonic waves at a frequency of 40 kHz and with a power of 500W. The resulting mixture was then centrifuged, and the supernatant removed to obtain the regenerated nanoparticles. The nanoparticles were rinsed three times to remove alcohol, free surfactant, acid, vinegar, and air dried. Fig 2 shows the synthesis schematic route.

### Yield of the nanoparticles after synthesis

The yield of the nanoparticles was calculated using the equation;

$$Yield\;(\%)=\frac{W_{a}}{W}\times 100\%$$ (1)

whereas *w*_*a*_ is weight of dry samples after synthesis and *w* is weight of dry samples before synthesis.

### Morphology

The morphology of the okra was analysed using Zeiss (LEO) 1450VP scanning electron microscope (SEM). The sample was placed on a slide with two-sided carbon conductive tape and a sputter-coated with gold. The sample was viewed and photographed at an accelerating voltage of 13 kV and a magnification of 500x. The size of CNP was determined using transmission electron microscopy (TEM). The analysis was done using HITACHI (Model:HT 7700). The sample was prepared by dissolving in DW and then placed on a platinum-coated microscopy grid. The stained specimen was observed with an accelerating voltage of 120 kV high resolution.

### Particles size analysis and surface charge

The hydrodynamic particle size and zeta potential were measured using a DLS litesizer 500 Anton Par equipment. The sample was prepared by dispersing the okra and CNP in DW at a concentration of 0.1% (v/v). The sample was then transferred to omega cuvette for measurement. The measurement was done at 25°C at a fixed backscattering angle of 170 degrees. The refractive index (RI) and the viscosity of water were 1.3303 and 0.8903 mPa.s, respectively.

### Crystallization structure variation

The X-ray diffraction (XRD) patterns of the okra and CNP were measured using a Rigaku SmartLab, Japanese diffractometer. With a CuK-beta radiation at 40 kV and 30 mA. The scattered radiation was detected at the 2θ angle scanning ranges of 3 to 50° at a speed of 8.2551°/min. The relative crystallinity was quantitatively calculated according to the following equation:

$$RC(\%)=\frac{Ac}{Aa+Ac}$$ (2)

whereas *Ac* is crystalline area and *Aa* is amorphous area on the X-ray diffractogram.

### Chemical structure and surface properties

The Fourier-transform infrared spectrometry (FTIR) spectra of the okra and CNP were determined using Shimadzu IR Tracer-100. The potassium bromide (KBr) FTIR method was used in this study. The dry sample was mixed with KBr before placing in the sample holder. The sample was then pressed into a disc before analysis. The FTIR spectra were performed within the wave ranges of 4000–370 cm^-1^.

### Preparation of CNF, okra and xanthan solutions

Different concentrations of CNF, okra, and xanthan (750–2000 ppm) were dispersed in DIW. The different solutions were homogenized, stirred for three hours and ultrasonicated for 30 minutes as previously described, this is to form a stable solution. To study the effect of salinity on the properties of the solutions, 0.2 wt% of the solutions were prepared in different synthetic brine solutions (0.9–2.2 wt%), these salinity concentrations range represent the Malay Basin oilfield salinity [3, 15]

### Viscosity measurement

The rheology of the CNF, okra, and xanthan was analysed using the rheometer (350 RST BROOKFIELD) installed with a temperature control and a connection for easy spindle attachment. The rheological evaluation was through a chamber designed to accommodate the coaxial cylinder spindle. The chamber has a rough surface to prevent slippage effect. The viscosities of okra and CNF under the different shear rates (1–1000 s^-1^) were measured at 26–80°C. Standard oil and pure water were used to calibrate the rheometer.

### IFT measurement

The IFT between DIW, nanofluids, and West Lutong crude oil was measured using K20 Easy Dyne Kruss tensiometer (Kruss GmbH, Germany). The ring method was used to calculate the IFT of the CNF at different concentrations as a function of electrolyte concentrations and temperature (26–80°C) according to the equation;

$$\sigma =\frac{F}{4\pi R\;cos\theta }(0.725+\sqrt{\frac{9.075\;X\;10^{-4}F}{\pi^{3}.\Delta \rho .g.R^{2}}}-\frac{1.679r}{R}+0.04534)$$ (3)

whereas *σ* is IFT, *F* is force acting along the 3-phase contact line which is equal to the weight of the liquid meniscus above the plane of the fluid-fluid interface, Δ*ρ* is density difference, cos *θ* is surface wettability, *g* is acceleration due to gravity, *R* and *r* are outer and inner radii of the ring respectively.

### Oil displacement test

**Fig 3:**
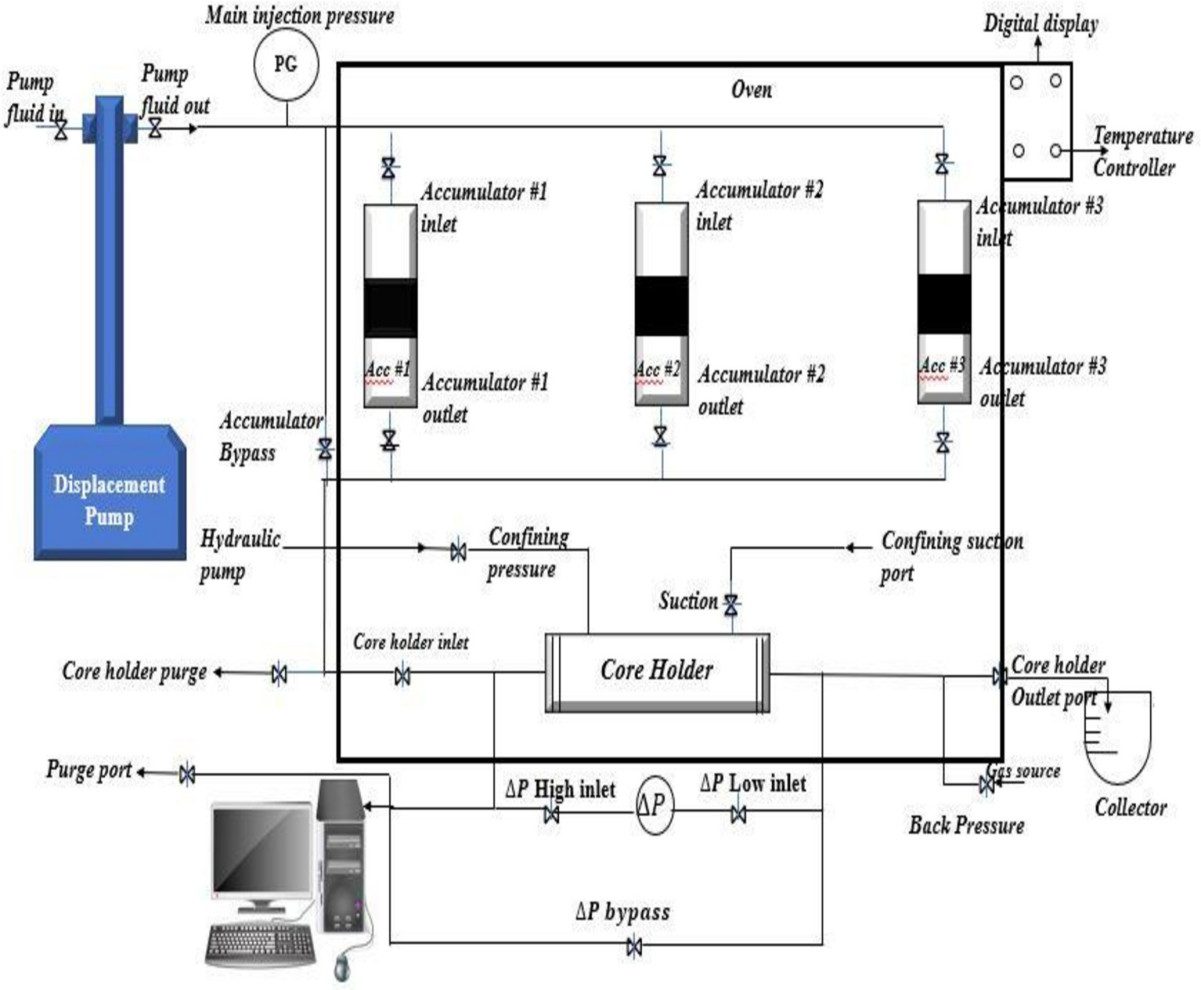
Schematics of the core-flooding experimental apparatus.

A high-temperature high-pressure (HTHP) core flooding equipment (Fars EOR Technologies) was used to determine the performance of the polymers and nanofluid at reservoir conditions. Fig 3 shows the schematics of the core-flood equipment. The apparatus is composed of fluid accumulator for brine, oil and chemicals, a back-pressure controller, core holder, a digital control oven and over-burden pressure. An ISCO displacement pump for liquid injection was used to pump liquid from the accumulator through the core-flooding system. A processor was linked to the equipment to control the flooding procedure, take the pressure, temperature and flow rate readings. The distillation extraction method in a Soxhlet column containing toluene was used to clean the cores. The cores were then dried at 100°C for 48 hours. To simulate a typical Sarawak oil field reservoir, the system was vacuumed, and subsequently pressurised to 3000 psi with a back pressure of 100 psi and the temperature of the oven was increased to 120°C. The core was saturated with 1.5 PV of synthetic formation brine (2.2 wt%), 0.5 mL/min of crude oil was injected into the core (to represent a laminar flow) until water production ceased, and the residual oil saturation was determined. The low injection rate was maintained throughout the experiment for proper sweep efficiency of the displaced fluid, which corresponds to nanofluid injection rate in an oil field [16]. The system was then aged for 24 hours to establish equilibrium between the liquid and rock surface to attain uniformity. Water flooding was performed and sustained until oil cut was less than 1%. EOR commenced by injection of 0.5 PV containing 0.2 wt% of xanthan or CNF to recover the residual oil. All the experiments were conducted at reservoir temperature (120°C) and pressure (3000 psi), except where otherwise stated. The flooding experiment was repeated thrice, and the average value was reported.

## Results and discussion

### Nanoparticle yield

**Fig 4:**
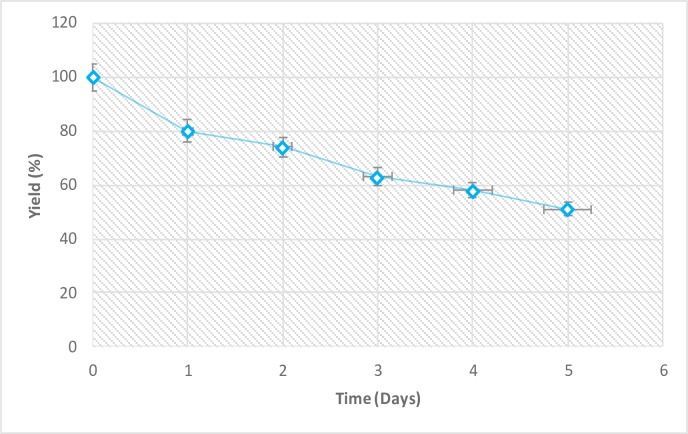
Yield of CNP after synthesis.

Fig 4 shows the yield of the CNP after synthesis. The yield of the CNP decreased with increase in synthesis time. The yield of CNP reduced from 100% to 51% after five days of synthesis. The decrease in yield is due to the effect of the weak acid hydrolysis on the okra mucilage. This could be because of the weak acid in plants extract used in this study. In contrast, a higher yield was recorded compared to the previous study by Shahrodin et al. [10] who used hydrochloric acid, which might have eaten up all the starch granules. The higher yield could also be attributed to the effect of ultrasonic, which would agree with the previous study of Kim et al. [13], who reported that ultrasonic treatment during acid hydrolysis of starch was effective in producing starch nanoparticles. During the synthesis process, the resulting nanoparticles aggregated to form sediments of microparticles. Nanoprecipitation retarded this aggregation and dissociated the nanoparticles [13]. The ultrasonic treatment dispersed the CNP, and changes in the sizes of the nanoparticles were examined by TEM and DLS. The amplitude of the ultrasonic increased the intensity of the bubble collapse and the disaggregation of the nanoparticle clusters became very effective.

### Morphological, chemical composition and structural change

**Fig 5:**
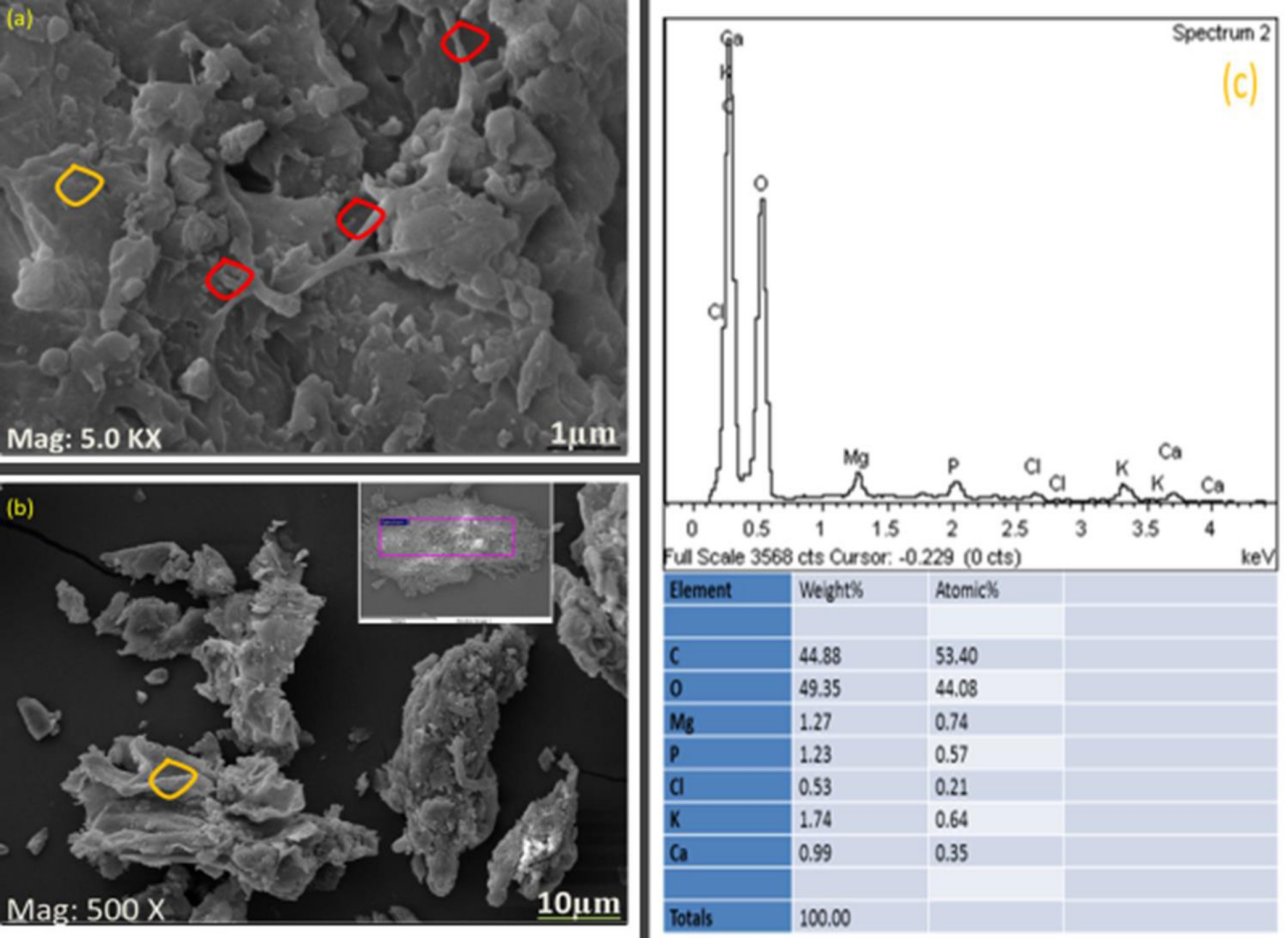
Image of okra (a) showing fibre bundles, (b) cellulose material, and (c) EDX spectra of okra.

The SEM image of okra at a magnification of 5000X and 500X were obtained (Fig 5). The images show an irregular, rough and compact arrangement of particles, which is consistent with previous studies of Zaharuddin et al. [17], who reported a dense and compact material with heavy cross-linking of molecules. Fig 5A shows stretching of fibre bundles (indicated in red) covered by cellulose materials (indicated in orange). The diameter ranges from 1–10 μm, which is consistent with the previous study of De Rosa et al. [18]. They reported that the cell wall thickness and lumen diameter vary between 1–10 μm and 0.1–20 μm, respectively. The difference in diameter of a single fibre and lumen of the okra combined with the rough shape of the okra was responsible for the mechanical and dimensional properties of the okra fibres. Fig 5C illustrates the EDX spectra of okra, the major elements appear at 0.1–0.5 keV whereas minor elements appear at 1.3–4 keV in the EDX spectra. The emission of strong signal belongs to carbon (C) and oxygen (O), which is consistent with previous studies of Acikgoz et al. [19]. They reported minor elements of Mg, P, Cl, K and Ca between 1.1–3.9 keV in the EDX spectra. This suggests that the relative atomic mass ratio of okra is well matched with the stoichiometry in preparation.

**Fig 6:**
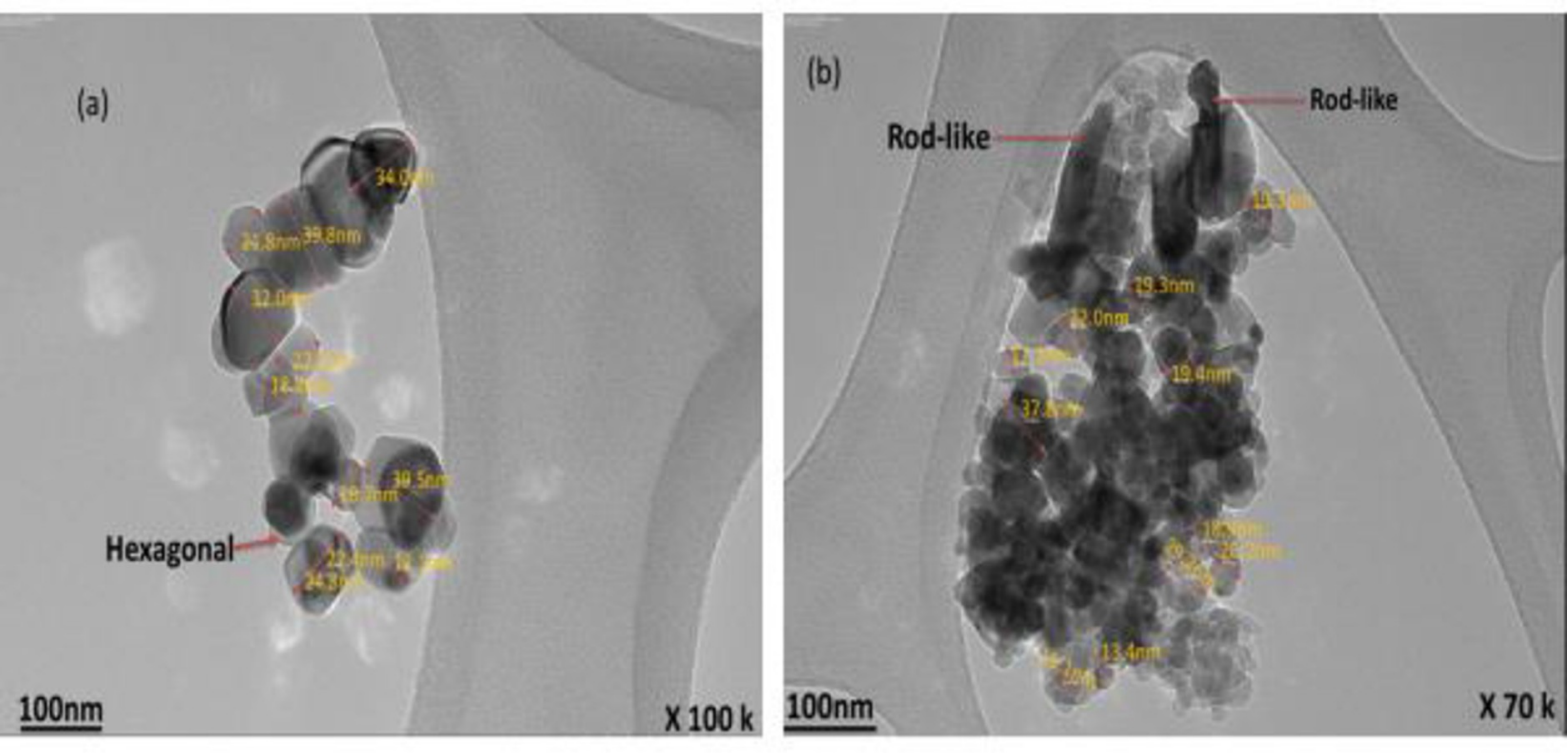
TEM image of CNP.

The changes in morphology are important to predict the interaction of the nanoparticles in solution. The morphology of the CNP was carried out using TEM to evaluate the structure and size of the particles. A reduction in the size of the particles occurred, and the particles appear separated into an individual nano-sized structure (Fig 6) with a variation compared to the original dimension of the okra (Fig 5). This due to the ultrasound treatment, the ultrasound improved the chemical reaction initiated and decreased the diffusion layer thickness initiated by ascorbic acid [12, 20]. The energy of the ultrasound is transferred to the okra particles by acoustic cavitation, producing penetrating microjets that brought the particles in a strong collision. This caused the disintegration of the okra granules into their smallest constituents. The energy of the ultrasonication weakens the cohesive force of the okra granules, making it easier for their detachment. The process continues inside the granules and cellulose chains are released in the form of tiny nanoparticles [21]. The nanoparticles appear well individualized with hexagonal, platy and rod-like shapes. The sizes range from 12.1–39.5 nm with a mean diameter of 100 nm. The mechanism responsible for the nanoparticle formation is a combination of two acoustic phenomena; emulsification and cavitation. Ultrasonic emulsification creates microscopic dispersion of the okra solution to form nanoparticles. The stability of these particles comes from the sonochemical cross-linking of the CNP [12]. During ultrasonic irradiation of liquid-powder slurries, cavitation and shock waves can accelerate solid particles to high velocities. This results in an interparticle collision that can induce striking changes in the surface morphology, composition, and reactivity of the nanoparticles.

### Particles size analysis and surface charge

**Fig 7:**
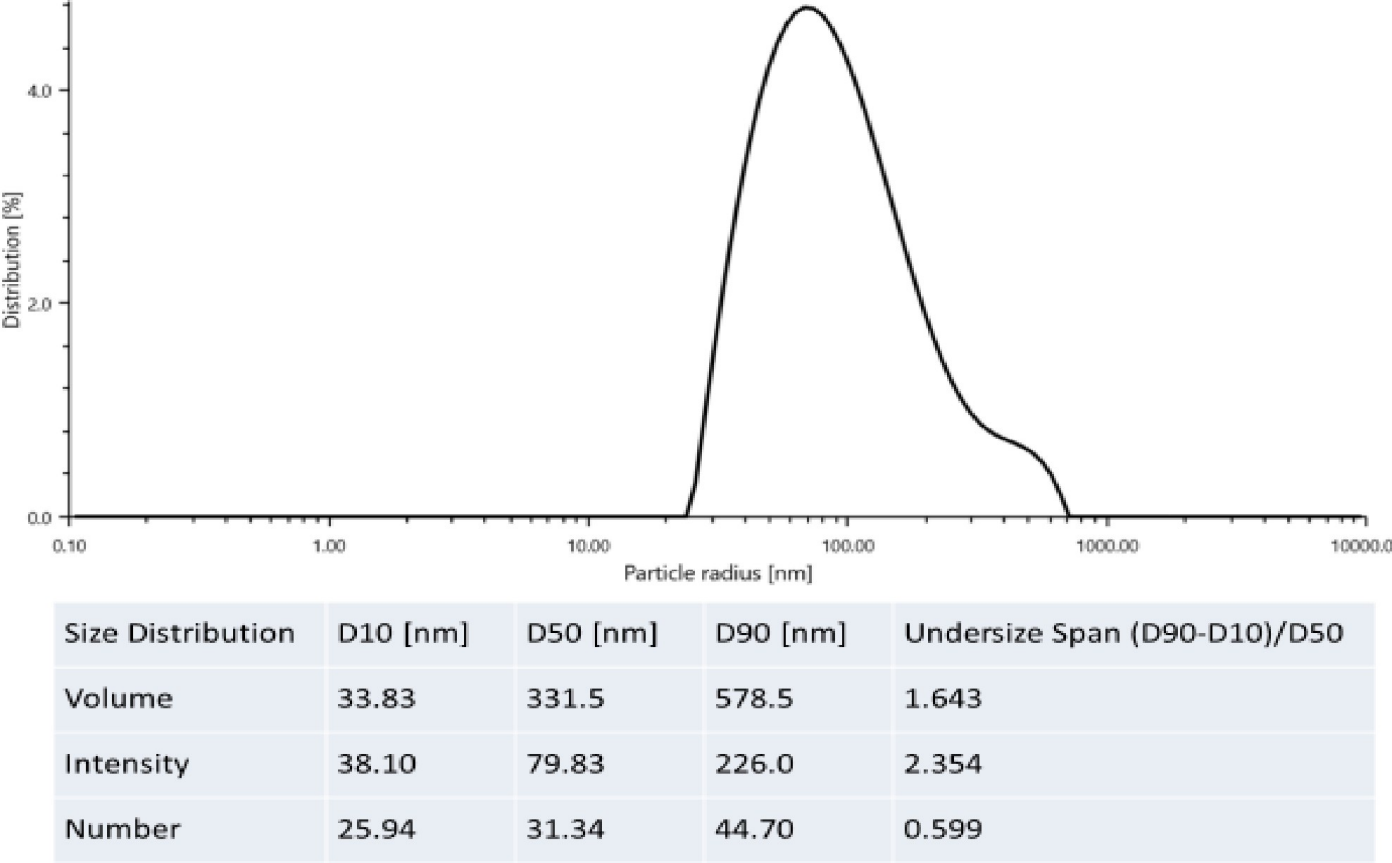
Particle size distribution by the intensity of CNP.

The sizes of the scattering objects are illustrated in Fig 7. The fluctuations of the scattering light that are a consequence of Brownian motions of the scattering objects that were analyzed. It shows that one major type of particle was present, a slowly moving pectin aggregate. The size of the slowly moving particles was around 117.5 nm. A similar result was reported by Sengkhamparn et al. [22], who observed a fast-moving object presumably pectin oil around 20–30 nm and a slow-moving pectin aggregate of 175–210 nm. The change in structure from micron to nanometer led to the emergence of coalescence. The interplay between Oswald ripening and coalescence through the molecular permeation theory could be the mechanism responsible for the change. Also, the exchange of molecules with droplet collision could be responsible. Such destabilizing mechanisms are usually reported for biopolymers where the mechanism is referred to as Oswald ripening-induced coalescence [23].

**Fig 8:**
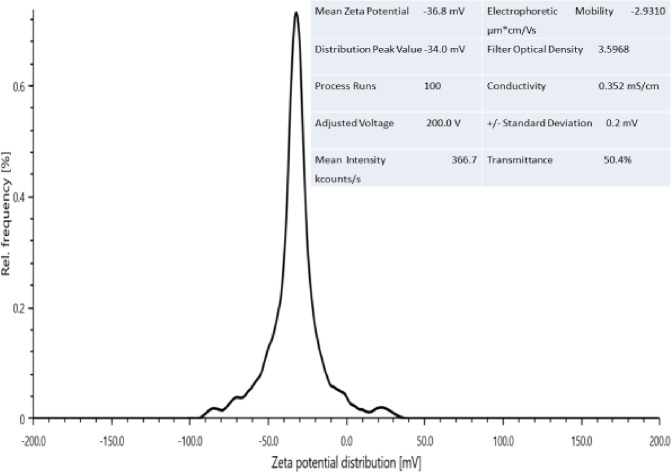
Zeta potential distribution CNP.

Zeta potential is an important parameter for determining the dispersion stability of colloids, indicating the degree of stability between adjacent similarly charged particles in the dispersion. For particles that are very small, a high zeta potential will mean stability. That is, the solution or dispersion will resist dispersion. Colloids with high zeta potential (negative or positive) are electrically stable while those with low zeta potential tends to coagulate or flocculate. Zeta potential measurement indicates the surface charge present on the nanoparticles when it exists in suspension [24, 25]. Fig 8 shows the zeta potential distribution of CNP. The mean zeta potential of -36.8 mV shows high electron charge on the surface of the CNP. It describes strong repellent forces among the particles which prevented aggregation leading to the stabilization of the CNP. The shift in zeta potential value from the original value of the okra (data not shown), might be attributed to the blockage of the active sites by the adsorption of the CNP chain [24]. As a rule of thumb, particles dispersion with zeta potential values of ±10 mV, ±10–20 mV, ±20–30 mV, and ±30 mV above are classified as highly unstable, relatively stable, moderately stable and highly stable, respectively [26]. This shows that the CNP is very stable and the mechanism in play is the electrostatic repulsion between the nanoparticles. This is due to the balance of the attractive versus repulsive forces that determine the stability of the nanoparticles systems. If the repulsive force is greater than the attractive force, the suspension becomes stable.

### Crystallization structure variation

**Fig 9:**
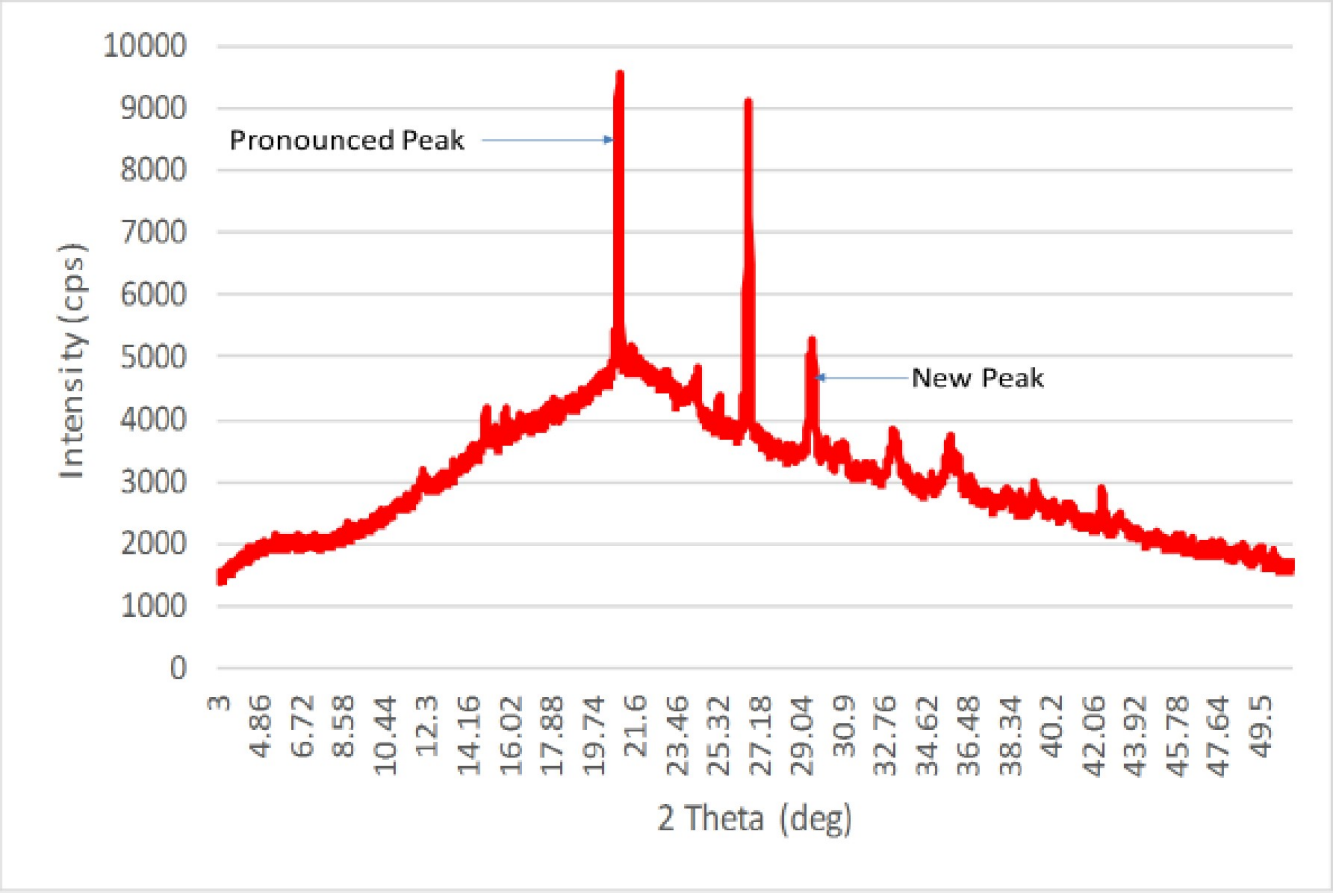
XRD diffraction of CNP.

The XRD of CNP is shown in Fig 9. The intensities of the peaks became more pronounced compared to the okra polymer. The RC of the CNP is 23% compared to 21% of okra. The increase in RC of CNP is because the amorphous region of the okra was removed by the weak-acid hydrolysis. The closeness in RC of both CNP and okra is the result of the strong organization of the okra, as seen in the SEM result (Fig 5). This is consistent with the previous study of Singh et al. [27], who reported that the proneness of potatoes starch to rapid changes in crystallinity signifies its weak organization. This increase in crystallinity could be attributed to the nanoprecipitation. The nanoprecipitation contributed to increasing the crystallinity of the CNP, which agrees with previous study of Qin et al. [28], who reported increase in RC during nanoprecipitation of high amylose corn starch nanoparticles. The precipitating medium preserved the crystallinity (23%), this could be because of the formation of a helical structure as the alcohol was added resulting in higher crystallinity [28]. This might be attributed to the rearrangement of the crystalline region of the fibre. The appearance of new and pronounced peaks at 2θ = 29.5°, 33.2° and 35.5° signal the introduction of cellulose II. This agrees with previous studies of Ouajai and Shanks [29] when they observed a crystalline transformation to cellulose II after treatment of hemp fibres. The transformation to cellulose II is a result of regeneration and mercerization of the cellulose-based material. This occurs where the chains are stacked forming corrugated sheets bound together by hydrogen bonding network.

### Chemical structure and surface properties

**Fig 10:**
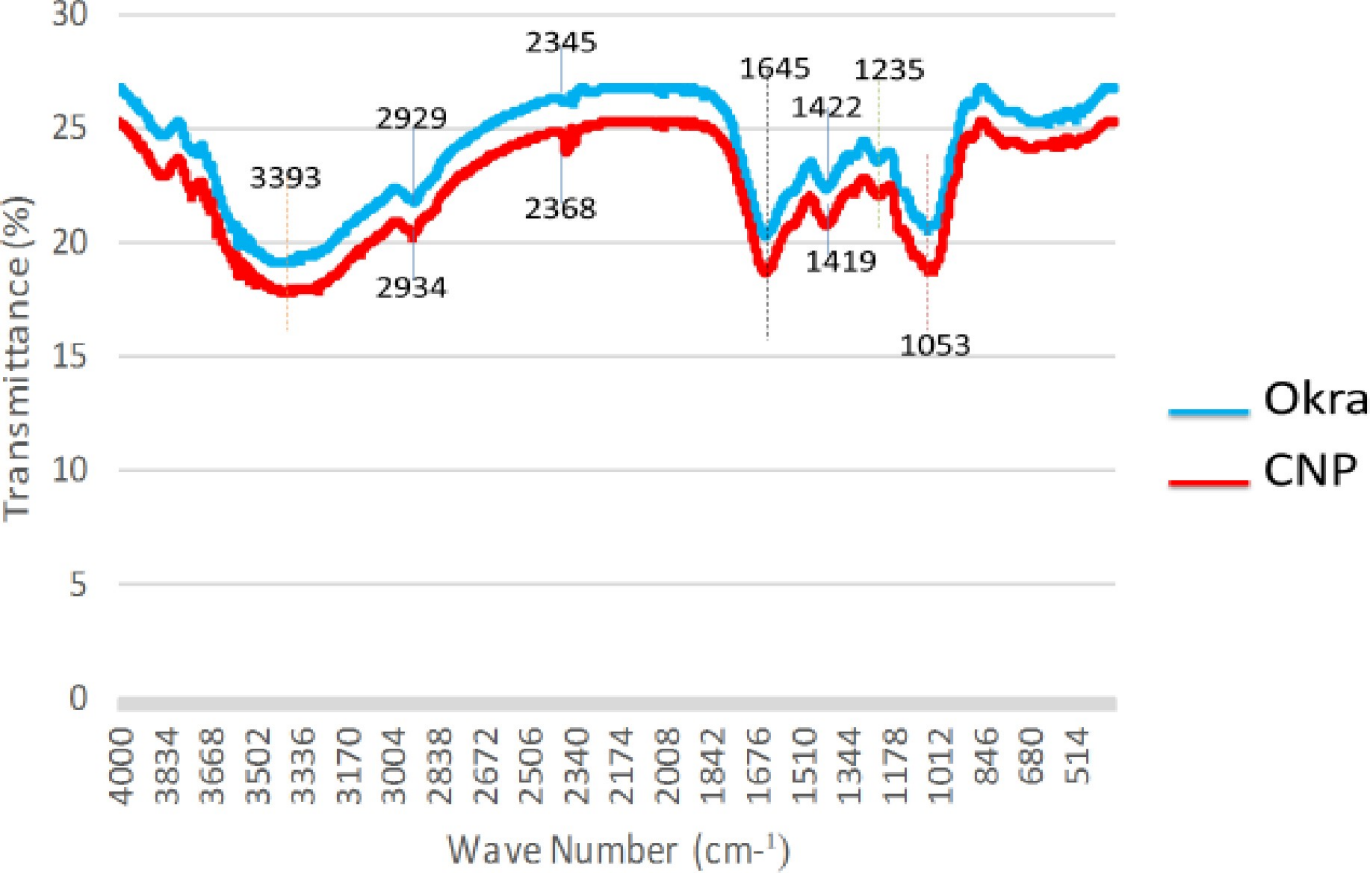
FTIR spectra of okra and CNP.

FTIR spectra were used to assess the structure of the okra and measure the change in surface composition of CNP after synthesis. The spectrum of the okra is similar to that of the CNP (Fig 10). A broad band between 3600–3100 cm^-1^ with the centre at 3393 cm^-1^ correspond to the O-H vibration and hydrogen bond of the hydroxyl group [30]. The peak at 2929 cm^-1^ is the band for C-H vibration from CH and CH_2_ in cellulose and hemicellulose [30]. The shoulder at 2345 cm^-1^ arises from stretching vibration of C≡N. The peak at 1645 cm^-1^ maybe due to the presence of water in the fibre. The peak at 1422 cm^-1^ is associated with the CH_2_ symmetric bending present in the cellulose. The band at 1235 cm^-1^ corresponds to the C-O stretching vibration of the acetyl group in hemicellulose compound. The sharp peak at 1050 cm^-1^ is ascribed to the CO and OH stretching vibration, which belongs to polysaccharide in cellulose and cyclic alcohol that is present in the natural polymer. This agrees with the previous study of De Rosa et al. [18], who reported that C-O stretching of the acetyl groups confirms the acetylation of the synthesized okra. Although both spectra look similar, there are some differences and the main spectra differences are attributed to the increase in the intensity of the symmetric in-phase ring stretching at 2919 cm^-1^ and 2345 cm^-1^ band to 2934 cm^-1^ and 2368 cm^-1^ respectively. These are a result of the in-phase angular deformation of the O-H group. Also, there is a decrease in the 1422 cm^-1^ band to 1419 cm^-1^. This decrease in peak intensity could be attributed to alkaline deacetylation of the hemicellulose. Synthesis of the okra led to extensive delignification; however, the intensity at 1053 cm^-1^ did not show any significant change whereas a shift in peaks observed at 1419 cm^-1^ is related to the skeletal vibration of the aromatics. These observations show that, after synthesis, the crystalline structure of the fibres was changed from cellulose I to cellulose II [29], which is consistent with the XRD results (Fig 9).

### Flow behaviour of CNF at different concentration

**Fig 11:**
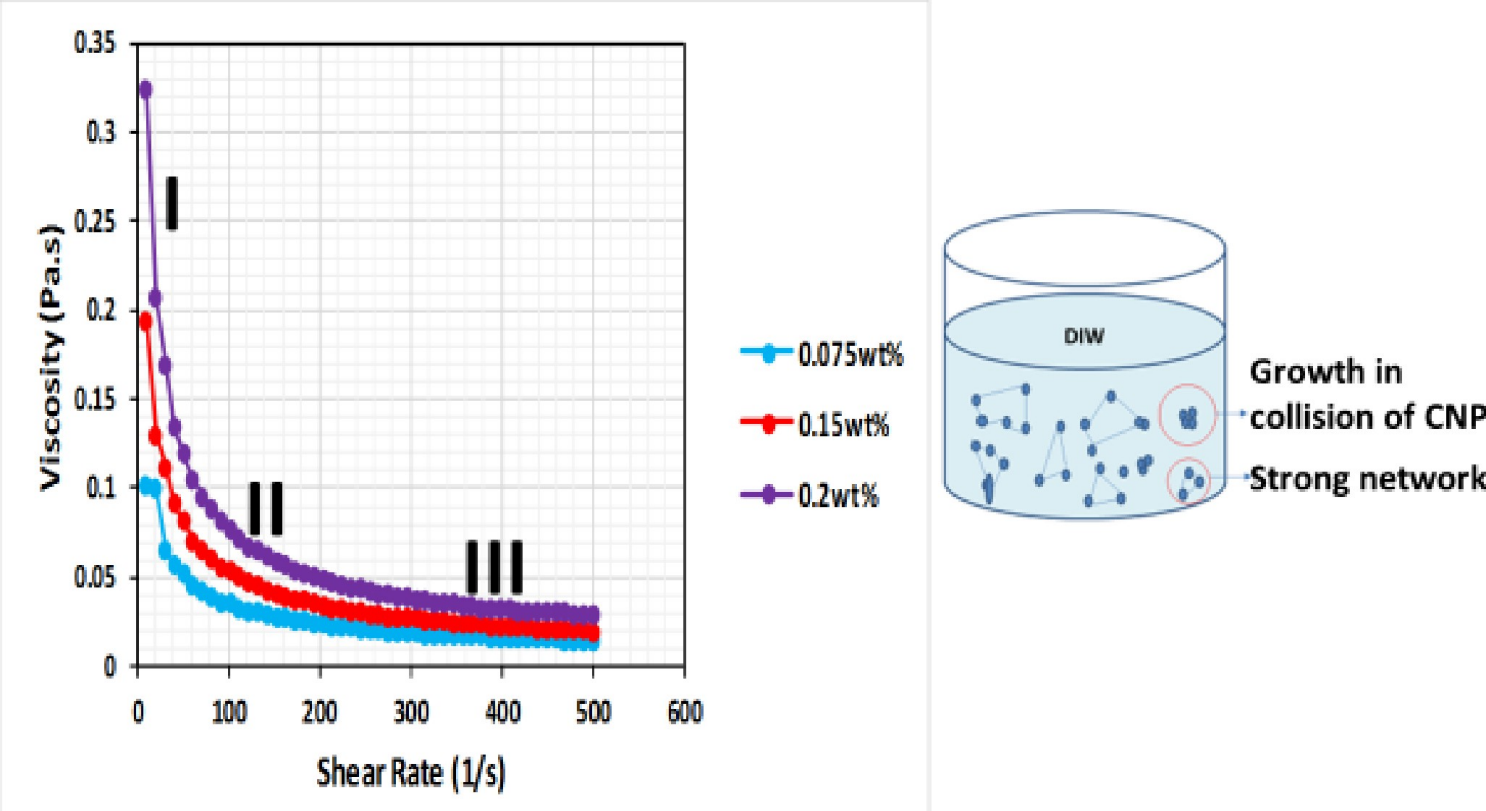
Apparent viscosity of CNF at different concentrations as a function of shear rate, showing the mechanism of shear-thinning effect.

The flow behaviour of the CNF was measured at 26°C (room temperature). The results show that the apparent viscosity is dependent on the shear rate at different concentrations (Fig 11). The apparent viscosity decreased with an increase in shear rate, which indicates that CNF is pseudo-plastic and exhibits a shear-thinning behaviour. The apparent viscosity increased with increase in the concentration of CNP. This shows that a strong network was created as the concentration was increased and there is a growth in the collision of the CNP [31, 32]. The shear rate curve can be divided into three regions. Region I; a steady decrease in viscosity was observed, this is due to the alignment of the CNP along the shear direction at a low shear rate [33]. Region II; all the CNP has been oriented along the shear direction and a decrease in viscosity is observed. This is because of the breakdown of the entangled network at an intermediate shear rate [32, 33]. Region III; a plateau is formed; this is because of the disruption of the entangled network at a high shear rate resulting in the formation of well-oriented structure which inhibited the decrease of viscosity [32, 33].

The shear-thinning region was fitted to Ostward DeWaele equation, *η = m*.*γ*^*n-1*^, where *m* is the consistency index, *n* is the flow behaviour index, *γ* is the shear rate and *η* is the apparent viscosity. Table 2 shows the *m* and *n* values of the CNF in the power region of *η* versus *γ* (*η = m*.*γ*^*n-1*^) as a function of concentration. The R^2^ values for the model are very close to unity indicating a perfect fit. Indicating that the power law model best describes the rheological behaviour. This signifies that the model is sufficiently accurate to predict the viscosity, at any given shear rate. It can be useful in the selection of optimum concentration, reformulation of nanofluid to a specification and to determine the shear-thinning behaviour of nanofluid in porous media. Shear thinning is indicated by *n*<1, all the concentrations show pseudo-plasticity and the pseudo-plasticity increases as the concentration increases.

**Table 2:** Consistency index (*m*) and the flow behaviour index (*n*) of CNF in the power region of *η* versus *γ* (*η = m*.*γ*^*n-1*^) as a function of concentration.

Concentration (wt%)	Consistency Index (*m*)	Flow Behaviour Index (*n*)	Stability Index (*R*^*2*^)
0.075	0.22	0.85	0.979
0.15	0.35	0.68	0.999
0.2	0.37	0.31	0.999

### Comparison of flow behaviour of cnf with xanthan gum and okra

**Fig 12:**
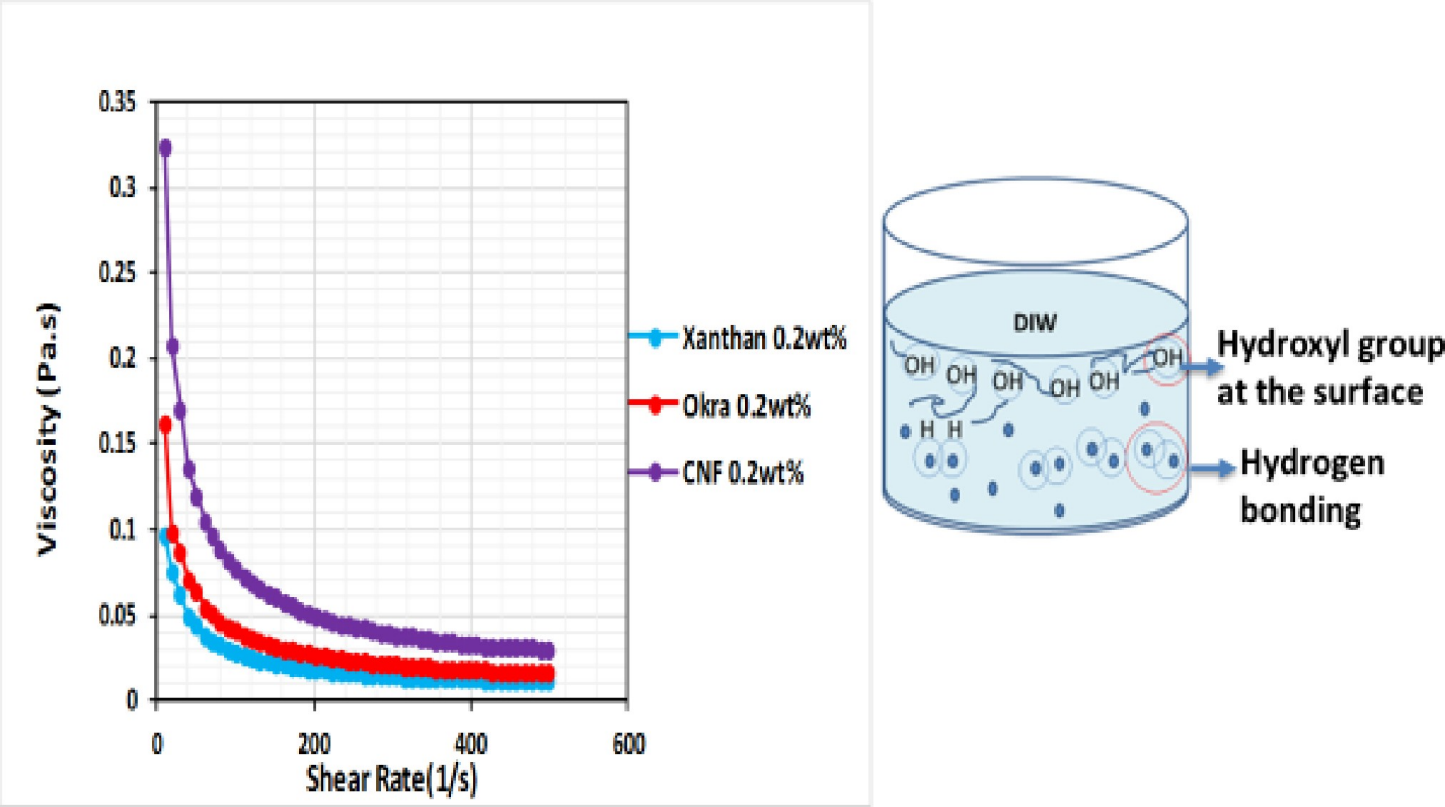
Apparent viscosity of CNF, okra, and xanthan as a function of shear rate, showing the mechanism of CNF viscosity reduction.

The apparent viscosity of CNF at room temperature and concentration (0.2 wt%) was determined and compared with that of okra and xanthan already in use in confectionary and the oil industry (Fig 12). All the samples show shear-thinning and the viscosity decreased with an increase in shear rate until it reached a plateau. This is because of the alignment of the particles, which greatly reduces their flow. At high shear, the chirality of the suspension breaks down in favour of a simple nematic structure [34]. The sample exhibited a pseudo-plastic shear-thinning power law fluid indicating that it is a yield stress fluid [34]. This could be as a result of the compact structure which inhibited the movement of the particles. The peak region in the flow curve is a characteristic behaviour of most lyotropic suspensions, where the particles are arranged in the direction of flow. The CNF showed higher viscosity at the same concentration (0.2 wt%) compared to the okra and xanthan. This could be because of the physical entanglement and inter- or intra-hydrogen bonding existing among the CNP, which accounted for the rheological behaviour. The physical entanglement between the okra chain was strong and highly flexible. However, the entanglement between the CNP chains was further enhanced after synthesis resulting in the formation of the hydrogen bond between the hydroxyl groups of the CNP. This led to higher viscosity of the CNF. Also, CNP has a larger surface area compared to the okra and xanthan. As such, more hydroxyl group were exposed to the surface of the CNP compared to the okra and xanthan, which enhanced the surface interaction. The growth of the collision between the CNP subdued the decrease of viscosity whereas the alignment of the polygonal okra and xanthan promoted the decrease of viscosity [32].

### Apparent viscosity of cnf, okra, and xanthan at different salinity concentrations

**Fig 13:**
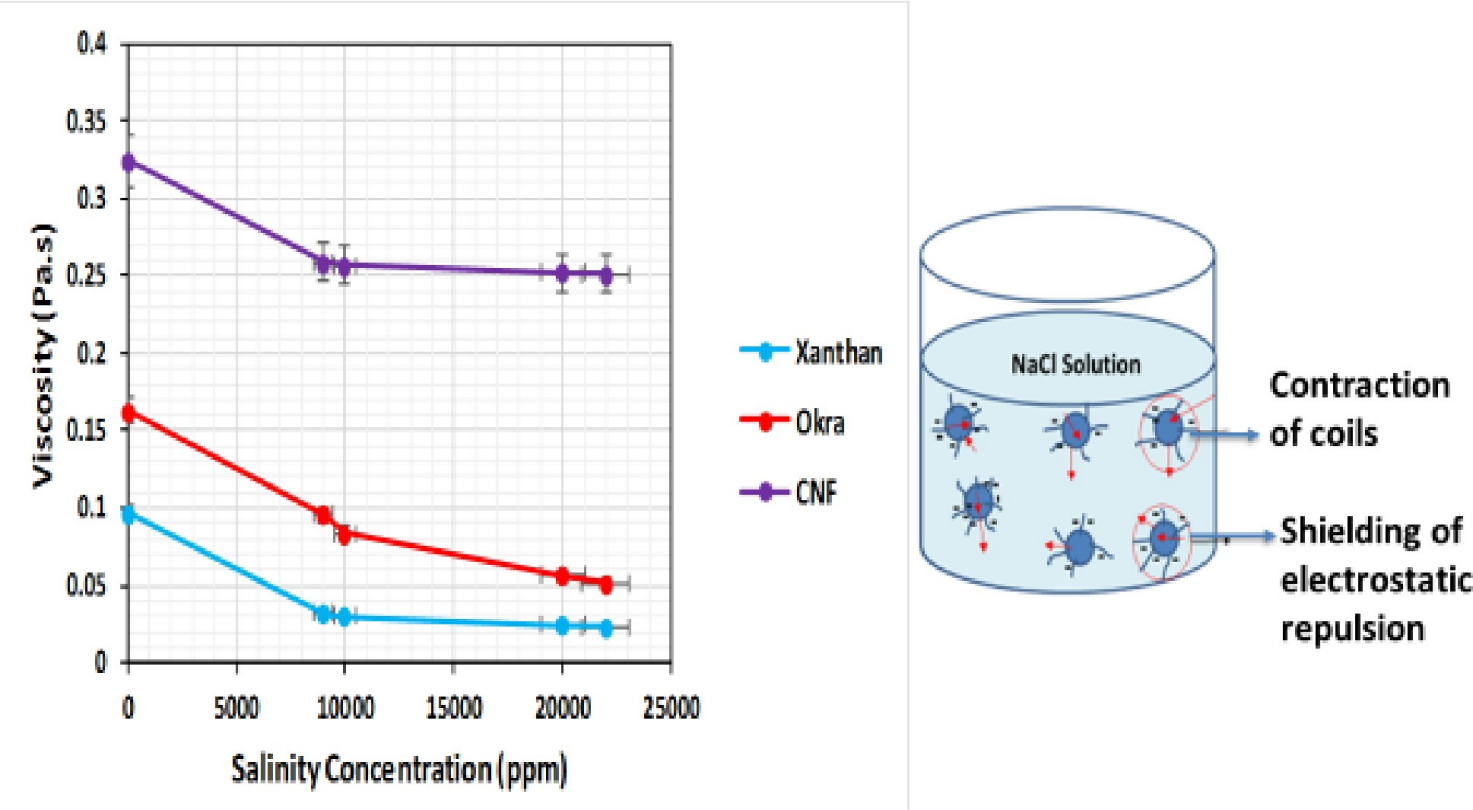
Apparent viscosity of CNF, okra, and xanthan as a function of salinity, showing the mechanism of CNF viscosity reduction.

The apparent viscosity of CNF at different salinity (0.9–2.2 wt%) was compared with that of okra and xanthan at the same concentration (0.2 wt%). It was observed that the apparent viscosity of all the samples decreased with increase in salt concentration (Fig 13). Such behaviour for CNP and okra is general for polyelectrolyte, as both are anionic polyelectrolyte. This is because, in water, the individual coils are expanded by intra-molecular electrostatic repulsion. The addition of salt screens the repulsion and allows the coils to contract to a more compact conformation, which led to the reduction in viscosity. This agrees with the previous study of Qiao et al. [35], who reported that increase in NaCl concentration (500 mM) led to the shielding of electrostatic repulsion, which weakened the interactions of cellulose nanocrystals (CNC) suspensions. However, the observed behaviour is inconsistent with the previous study of Agoda-Tandjawa et al. [36], who reported an increase in viscosity with increasing concentration of NaCl. The formation of gel in their studies might be responsible as salinity influences the high ionic strength of the suspension. This was not the case in this study; therefore, the flow properties are not sensitive to the addition of salt. This was more pronounced in the CNF compared to the okra and xanthan. This is because of the removal of lignin and hemicellulose from the okra during synthesis, which led to a strong repulsive hydration interaction resulting in the higher viscosity in the CNF. The increase in viscosity in the previous study of Agoda-Tandjawa et al. [36] might be due to electrostatic repulsion.

### Effect of temperature on apparent viscosity of CNF, okra, and xanthan

**Fig 14:**
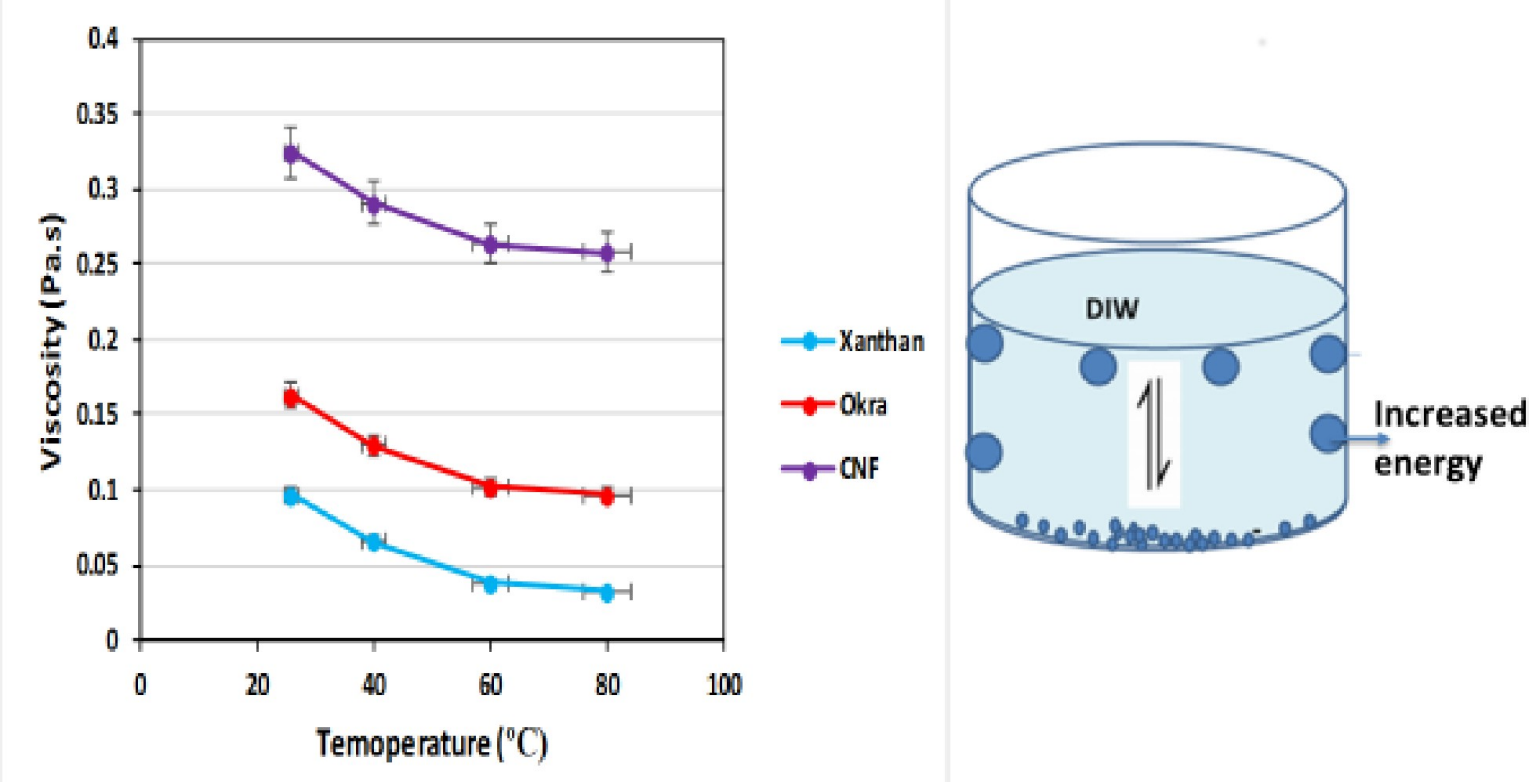
Apparent viscosity of CNF, okra, and xanthan as a function of temperature, showing the mechanism of viscosity reduction.

The effects of temperature (26–80°C) on the viscosity of CNF, okra and xanthan are shown in Fig 14. The results show a slight decrease in viscosity as the temperature increased. This could be due to increase in energy of the molecules, which led to a decrease in the inter molecular interaction [37]. The decrease might be due to the weakening of the inter molecular interaction in the suspension at high temperature. This agree with the previous study of Garcia-Ochoa and Casas [38]. They reported that the decrease of the apparent viscosity with temperature is reversible and it is due to the interaction of the molecules in solution, which became weaker at high temperature. However, for CNF the decrease was not pronounced because a high viscosity was maintained compared to the okra and xanthan. The difference in viscosity at the same concentration (0.2 wt%) might be from the difference in the microstructure of the solution, which is due to the enhancement of the associate forces between the CNP after synthesis. Although the temperature altered the viscous nature of the samples, it did not affect the pseudo-plasticity.

### Effect of concentration on IFT of CNF

**Fig 15:**
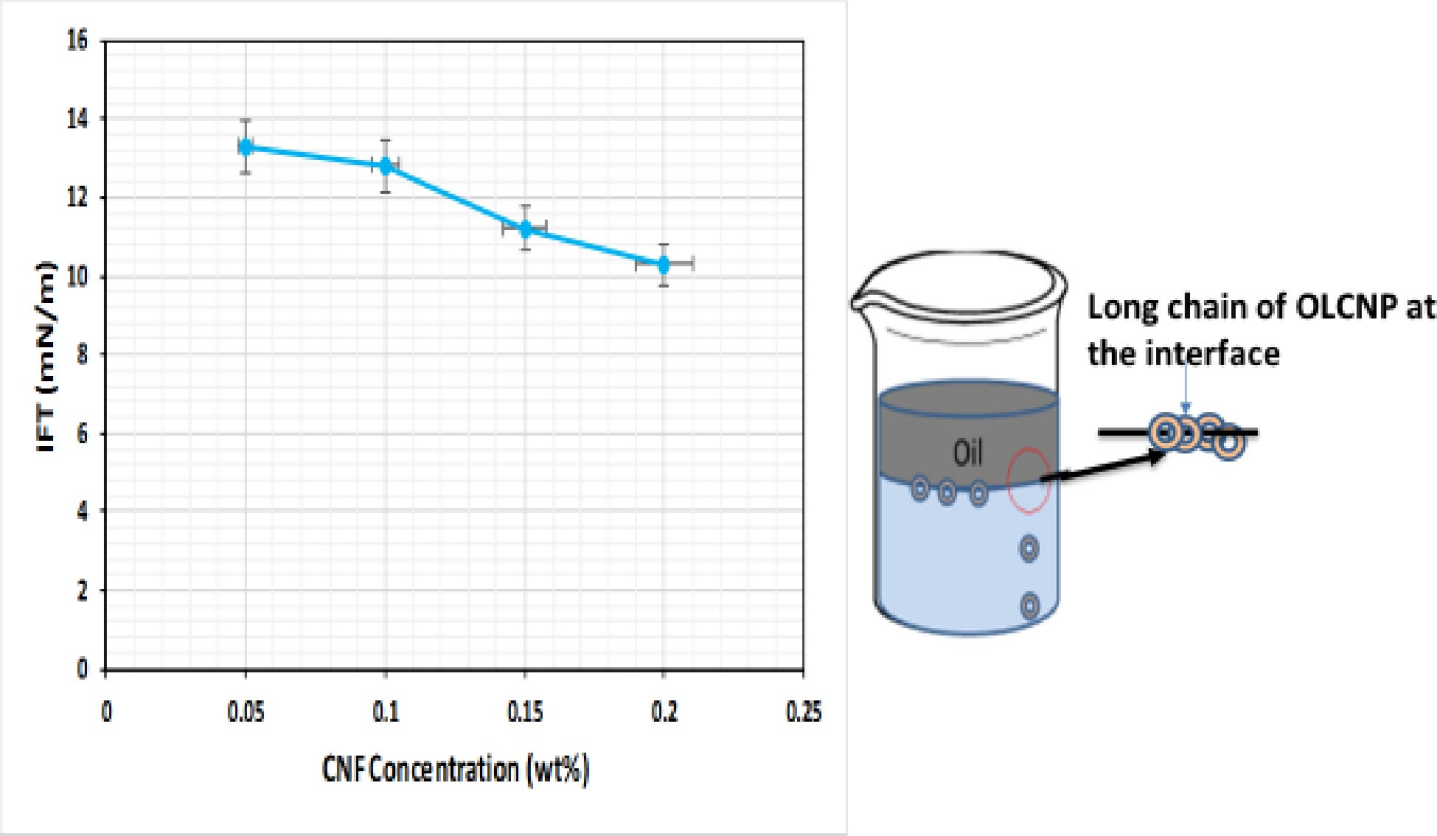
IFT of CNF (DIW) as a function of concentration, showing the proposed mechanism of IFT reduction.

Fig 15 shows the effect of change in concentrations (0.05–0.2 wt% w/v) on the IFT of CNF. The result shows the dependence of O/W IFT on CNF concentration. IFT decreased with increase in the concentration of CNF, which is consistent with the studies of Pei et al. [39], Ogunlaja et al. [40] and Ye et al. [41]. As the concentration of the CNP increases, more particles are driven to the O/W interface. The long chain of the CNF attached themselves to the surface and form a layer, which increases the potential between the particles and imparts a repulsive force between them and reduces the energy at the interface and IFT. The repulsive force was induced by an acid group of CNF that polarized the water whereas the thioglycolic group of the CNF induced an electrostatic interaction that synergistically reduced the IFT. Increase in concentration of CNP increased the interfacial area and reduced the energy at the interface that might have also reduced the IFT. This is because the ultrasonic reduced the surface area to volume ratio and increased the hydrophobicity of the CNF. This is in line with the study of O’Sullivan et al. [42] who reported that ultrasonic stimulation makes the interfacial film more hydrophobic. This enables the sonicated aggregate to adsorb faster to the interface forming an interfacial film, inducing steric and electrostatic interaction and thereby reducing the IFT. At high ultrasonic wave, compression and expansion enact stress to the interface overcoming the interconnected force that holds large droplet, breaking them to smaller ones. This increases outward motion and the hydrophobicity at the interface, resulting in the increased cavitational threshold. The cavitational threshold decreases cavitational activity; thus, the amplitude of the interfacial instability is reduced [43].

### Influence of electrolyte concentration on IFT of CSNF

**Fig 16:**
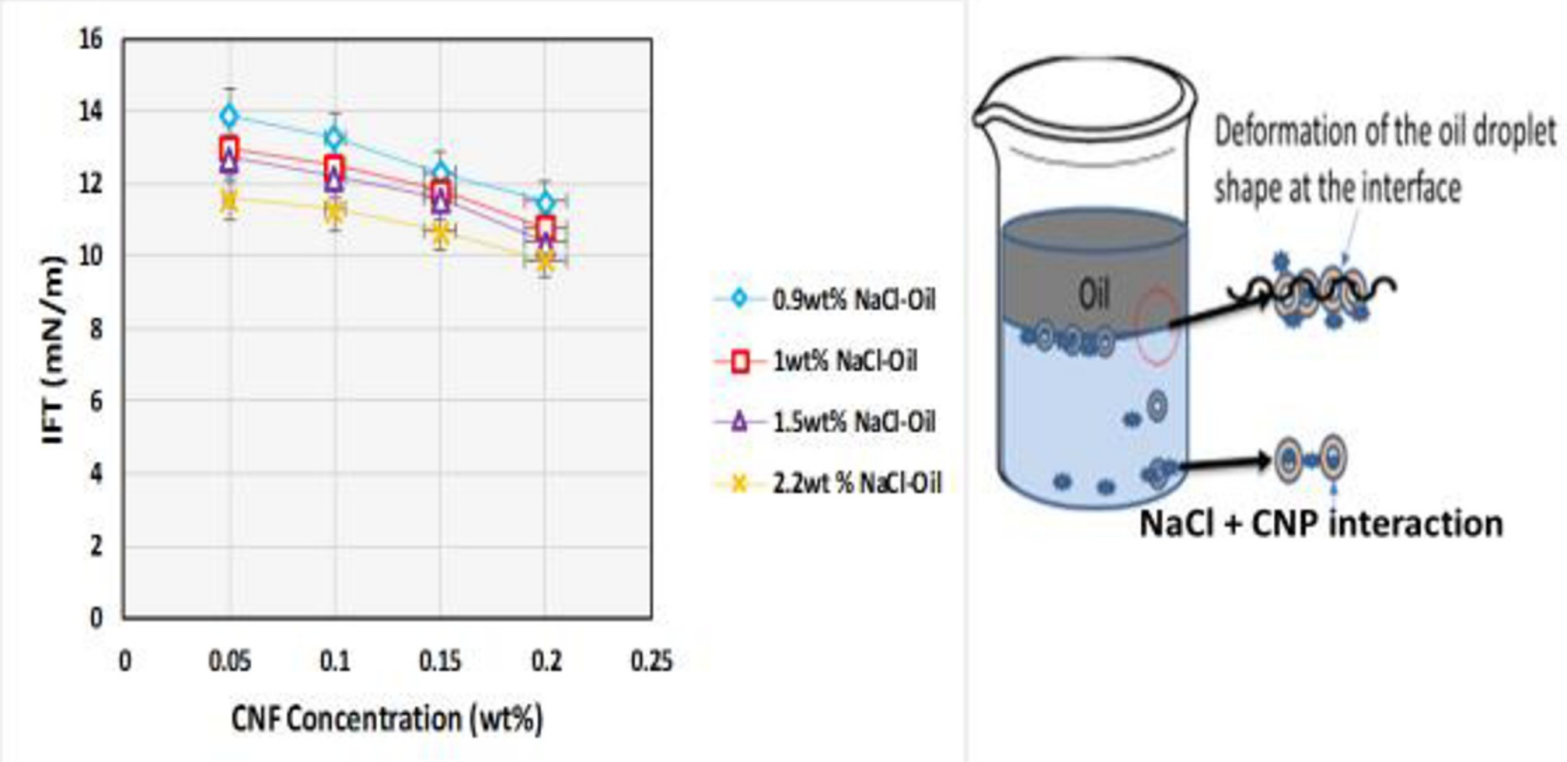
Influence of electrolyte on IFT as a function of CNF concentration, showing the mechanisms of IFT reduction in the presence of an electrolyte.

The IFT of crude oil and CNF with NaCl (0.9–2.2 wt%) is shown in Fig 16. The IFT decreased with increase in NaCl concentration. This might be due to the dissolution of the crude oil compound by NaCl and subsequent sorption of the amphiphilic compound at the interface of O/W. This liquified crude oil in the NaCl acted as a natural ionic and non-ionic hydrocarbon surfactant [44]. It might also be because of the synergetic influence of the NaCl and CNP on the IFT. The NaCl lowered the solubility of the CNP making it less ionized, which might have resulted in CNP adsorption on the O/W interface. The effect of ultrasonic was also felt in the aqueous suspension containing NaCl and CNP, to which the sonication was applied. As the NaCl produced dilute aqueous dispersion of the CNP that deformed the oil droplet shape at the interface which might have reduced the IFT. Ultrasonic increased the concentration of the surface-active agent formed by the addition of NaCl, which led to phase separation and decreased volume fraction of the chiral nematic and isotropic phase. Ultrasonic also hindered the formation of anisotropic phase formation in the CNP suspension and influenced the formation of a double electrode layer and reduced the layer at the interface [45]. This led to the CNF molecules arranging themselves at the interface, which increased the surface activity of the polar impurities. In the presence of NaCl, the impurities are salted out and the concentration at the O/W interface lowers the IFT [46].

### Temperature effect on the IFT of CSNF

**Fig 17:**
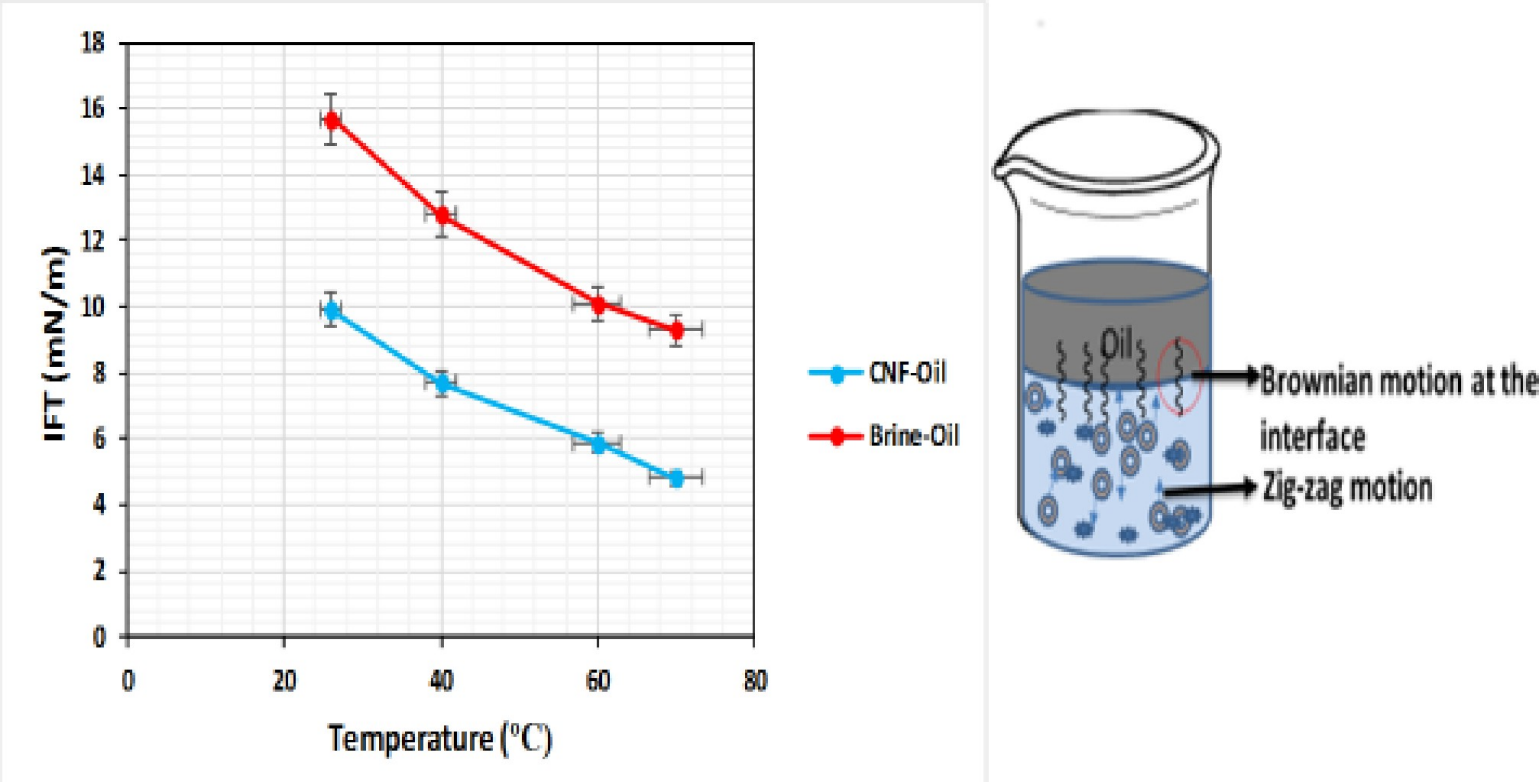
IFT of CNF (DIW) and electrolyte as a function of temperature, showing the mechanism of IFT reduction.

The effect of temperature (26–80°C) on the IFT of CNF (0.2 wt% w/v) is shown in Fig 17. The results show that CNF reduced the IFT by 52% compared to brine (NaCl 0.2 wt%) at 26°C. This, in turn, shows that IFT of CNF decreases with increase in temperature. This is because an increase in temperature of the CNF, increases the kinetic energy of the liquid molecules. During the ultrasonication of the CNF, cavitation played a vital role in percolation of phases exposed to ultrasonic. CNF molecules tend to migrate to the interface and coalescence occur. The coalescence of the CNF droplet is because of collision frequency of dispersed CNF, acoustic streaming, the attractive force acting between the oscillating droplet and heat generated during sonication (Bjerknes forces). This led to expansion and weakening of the intermolecular forces at the interface during the IFT test, thereby, reducing IFT. At higher temperature, there is an increase in the zig-zag motion of the particles (Brownian motion). The Brownian motion builds inside the molecule of the liquid; this will, in turn, reduce the interconnected energy and the IFT of the CNF [47]. It might also be due to the increase in solubility of O/W, an increase in solubility decreases the interfacial energy which lowers the IFT.

### Oil displacement results

**Fig 18:**
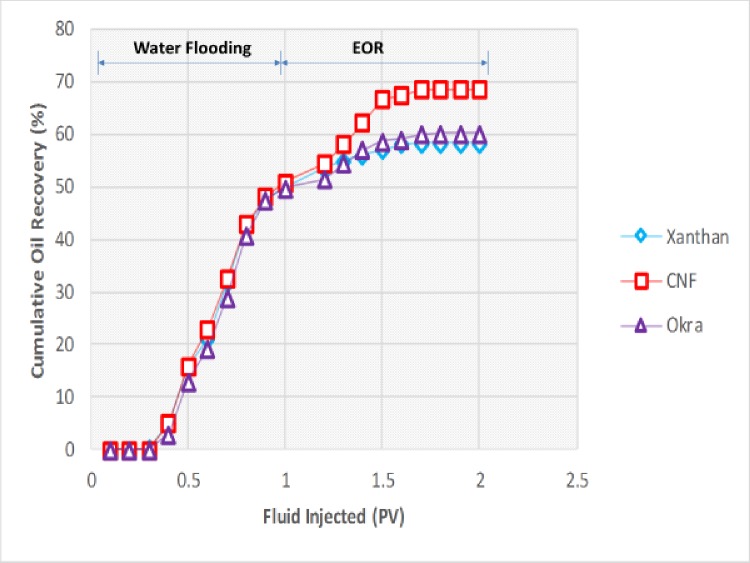
Cumulative oil production performance.

The performance of the okra and CNF were determined by laboratory scale core flooding. And the results were compared with commercial polymer xanthan used in EOR (Fig 18). The oil recovery after water flooding was 48% original oil in place (OOIP), this showed that a substantial amount of oil remained in the core. Tertiary recovery commenced immediately, and the oil recovery with okra increased by 13% compared to the initial 11% OOIP obtained with xanthan. The slight difference between the results might be due to its similar functional properties with xanthan [48]. As a result, the viscosity and other rheological properties of both okra and xanthan are stretched and become sufficiently close to each other [49]. The increase in recovery by xanthan flooding compared to the water flooding can be attributed to the blockage of the continuous water channel generated during water flooding. The high viscosity of xanthan reduced the mobility ratio which might have contributed to mobilizing the trapped oil [50]. The okra producing more oil compared to xanthan can be attributed to the improved stability of the flood by the okra. The ability of the okra to withstand degradation at high temperature and salinity might have improved its efficiency in banking oil through a favourable mobility ratio which might have improved the sweep efficiency [51]. This result is consistent with the previous study of Hatcher, [52] when they reported that biopolymer schizophyllan can retain its viscosity up to a temperature of 135°C. No loss of viscosity was found after 300 days at 110°C under anaerobic conditions and showed excellent laboratory and field trial results when compared with commercial polymer xanthan and sulfonated polyacrylamide. Therefore, the improvement of the okra flood can be attributed to the fractional flow behaviour of the okra [53].

It was observed that the oil recovery of CNF increased by 20% OOIP compared to the initial 11% with commercial polymer xanthan and 13% okra. The oil recovered by the CNF is higher compared to xanthan and okra, this is because CNF blocked the permeable zones and recovered oil from small pores [1]. The trapped oil droplets or ganglions are mobilized due to the reduction in the IFT between the O/W. It might also be due to the enhancement of the viscosity by the CNF which might have controlled its movement thereby, improving oil recovery. It can be concluded that CNF was effective in improving residual oil recovery and the recovery was not affected by the high temperature (120°C) and pressure (3000 psi) reservoir condition. Which is consistent with the IFT results as the IFT of the CNF decreased with increase in temperature. This confirms the role of IFT to recover residual oil.

Fluid displacement in porous media was controlled by viscous, capillary, hydrodynamic and gravity forces. A ratio between any two of these forces can be expressed as a dimensionless number [54]. Dimensionless numbers can be used to quantify the effect of these controlling forces in EOR process. In a normal flooding process, capillary forces dominate the microscopic displacement process whereas the microscopic distribution of oil and water is determined by the conditions of hydrostatic equilibrium. To assess the transition between a displacement process dominated by capillary and viscous forces, it is convenient to consider the dependence of recovery on a suitable dimensionless parameter such as capillary number;

$$capillary\;number\;(N_{ca})=\frac{\mu \nu }{\sigma }$$ (4)

whereas μ is fluid viscosity, ν is fluid velocity and σ is O/W IFT.

**Fig 19:**
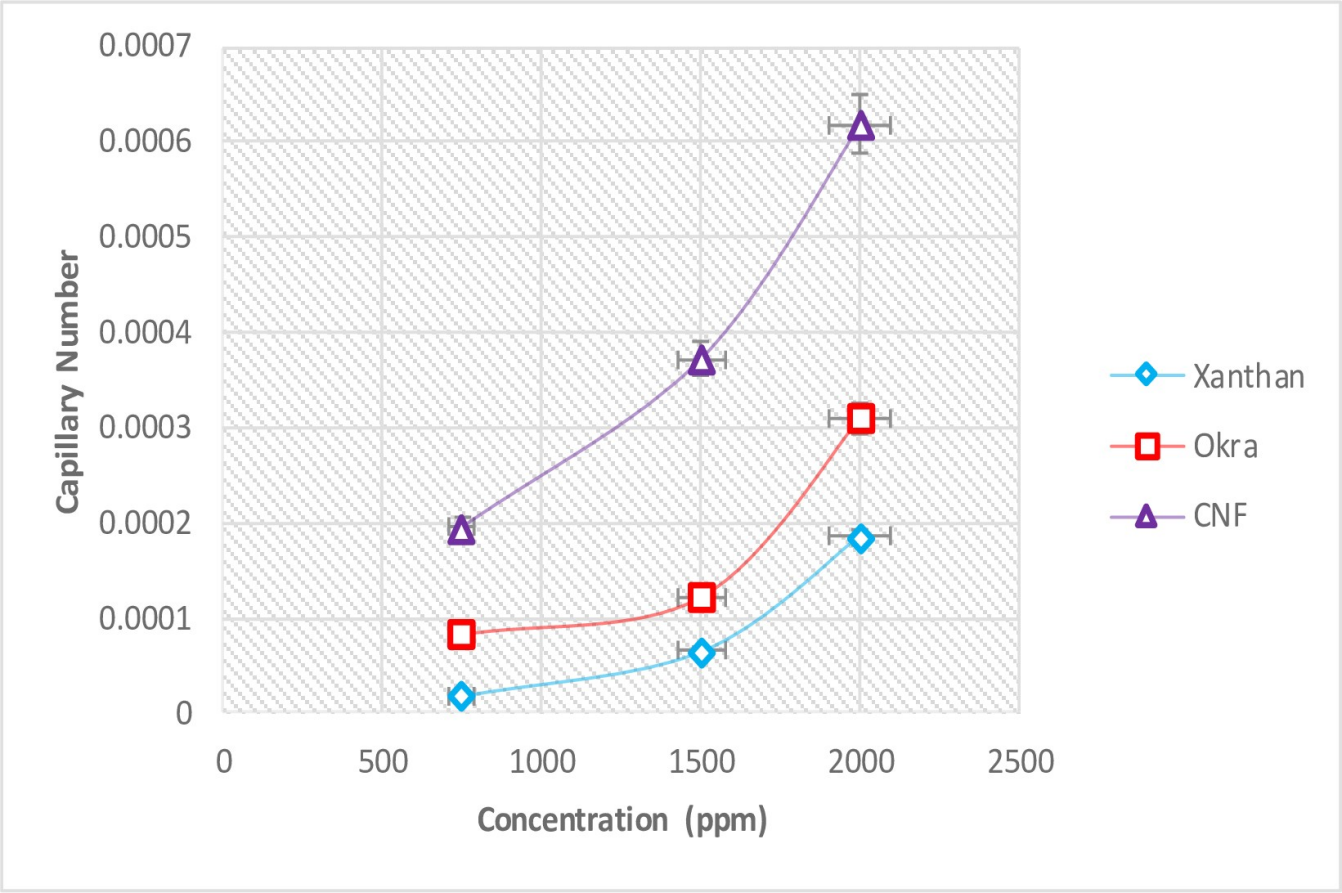
Effect of CNF, okra and xanthan on capillary number.

Fig 19 shows the effect of CNF, okra and xanthan on capillary number. The capillary number increased with increase in concentration for all the fluids. This implies that the viscous force is dominant over the capillary force, the capillary force was no longer sufficient to cause the flow [55]. The higher capillary number of CNF is due to its lower IFT compared to okra and xanthan. This is consistent with the IFT results, which indicates that the capillary number is a function of IFT. The increase in capillary number indicates that the oil flow has become faster through the pore throat, this is because capillary forces was reduced by increase in capillary number. This phenomenon increased the microscopic displacement efficiency of CNF compared to okra and xanthan as capillary number increased.

**Fig 20:**
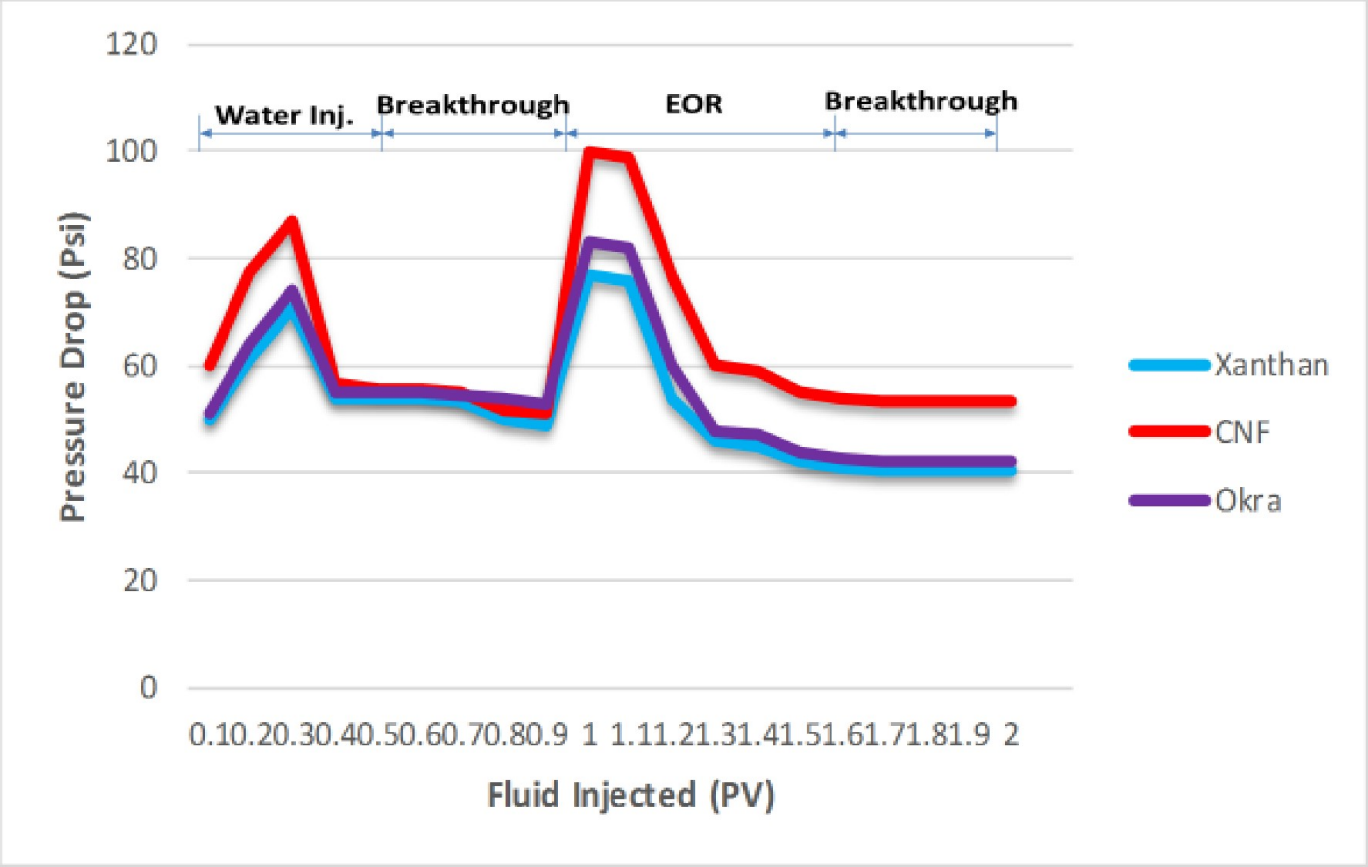
Pressure drop profile of xanthan, okra, and CNF as a function of fluid injected.

The oil recovery was validated by the pressure drop profile (Fig 20). At the beginning of the water flood, the pressure drops increased, it then fell sharply and continue to decrease gradually and was constant. The decline is because of higher movement of the water than the oil. The constant pressure drop reveals the breakthrough of water. The pressure drops increased during xanthan and okra injection which is because of higher viscosity of both xanthan and okra to water [1]. The rise in the pressure drop suggests that injected fluids interacted with the trapped oil which resulted in the dispersal of the oil into the aqueous phase and subsequent displacement from the core [56]. The pressure drop increase was higher for CNF compared to xanthan and okra. This could be attributed to the strong oil mobilization and the two-phase flow in the porous media. The high-pressure drop also demonstrates that the CNF was more stable at 120°C by maintaining the pressure drop during the flooding at high temperature. This shows that at 120°C, the oil bank formation was enough to increase oil recovery.

**Fig 21:**
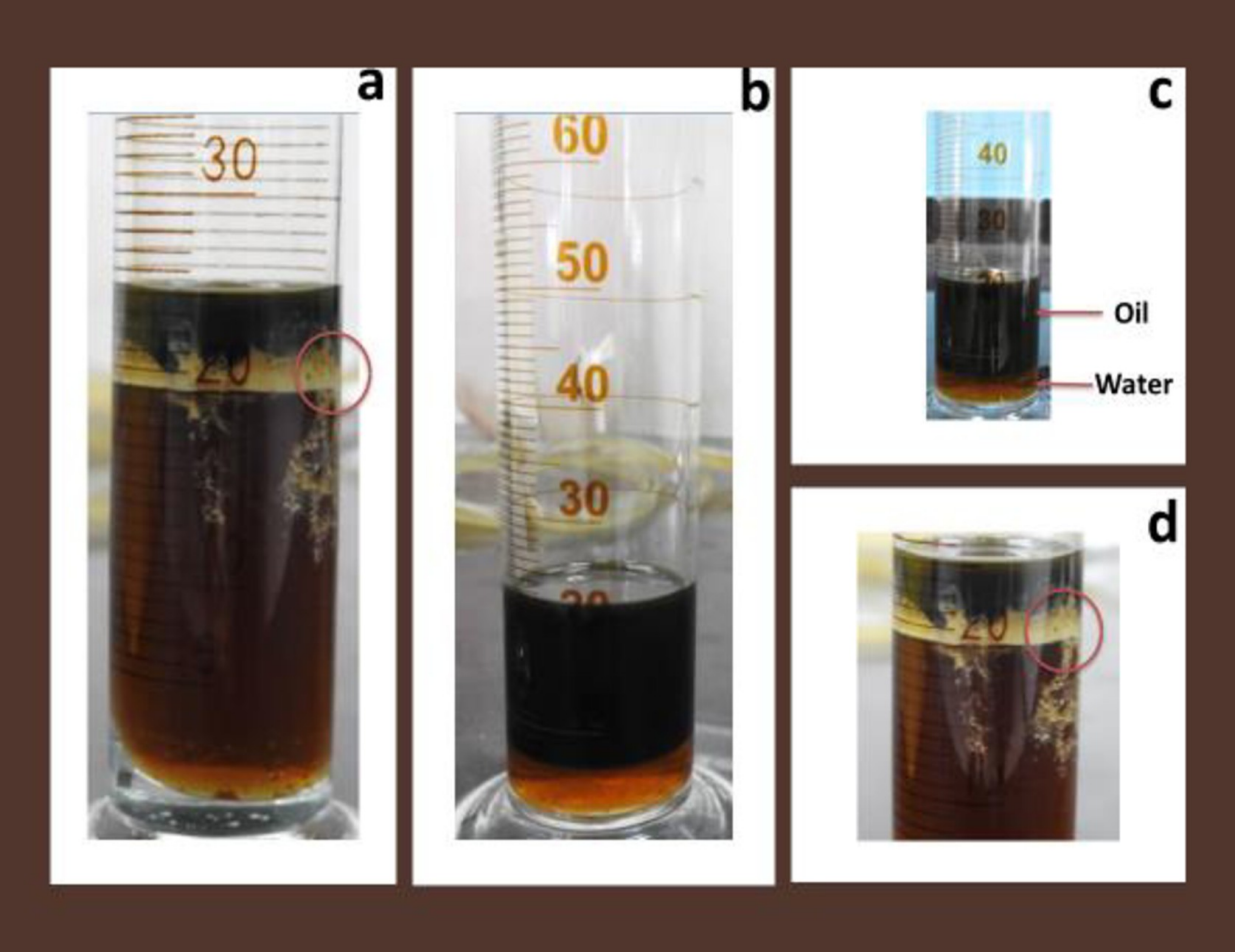
(a, d) CNF oil recovery showing emulsion formation (b, c) xanthan and okra oil recovery showing absent of emulsion.

The high peak value of the pressure drops resulted in high oil recovery. The build-up pressure is due to the penetration of the CNF into the remaining oil drops to form a high viscous O/W emulsion (Fig 21A and 21D). Which might have blocked the permeable water channel and reduced the movement of the oil phase resulting in improved sweep efficiency [50]. This is evident in the fluctuation in the trend of the pressure drop observed during CNF flooding as the emulsion formed can reduce the flow capacity of some channel formed during water flooding through pore blockage mechanism and divert the injected font as well as favourable mobility of the emulsion to displace oil banks [57]. This phenomenon might have enhanced the mobilization of the oil, as the film separated the oil from the formation rock and more oil was removed (21a, d) compared to xanthan and okra flooding where emulsion formation was not experienced (21b, c). Therefore, the emulsion formed under this condition improved the sweep efficiency by reducing viscous fingering and water channelling [58].

### Energy and cost estimation

The energy required for the synthesis process was calculated from the equation;

$$E=\frac{Pt}{R_{m}}$$ (5)

whereas, *E* is the required energy (*J/g*), *P* is the applied power (*J/s*), *t* is the ultrasonication time, *R*_*m*_ is the amount of treated raw material (*g*).

The energy calculated for this study was 3 x 10^4^ J/g, which is lower than the energy required for the steam explosion (9.90 x 10^4^), ultrasonic-assisted enzymic hydrolysis (4.16 x 10^4^) and other conventional methods [59, 60]. This is consistent with a previous study of Nitayavardhana et al. [61], who reported that the cost of energy consumption for ultrasonic pre-treatment of cassava chips to produce ethanol was 11 kJ compared to 22 kJ for heat treatment. The finding here also agrees with those in previous studies of Bubalo et al. [62] and Mullick and Neogi [63], who reported that ultrasonic provides an easy route to synthesize composite in less time and reduce energy consumption by more than 45–65% and 96%, respectively, compared to using conventional methods. Considering the capital and operating cost, a model plant would save approximately $1,114, 447 each year on pre-treatment cost if it invests in ultrasonic [64]. The total cost (equipment and raw materials) of this method is about $229, which is approximately 25% less than the cost of production design for manufacture and assembly analysis of photoelectrochemical (PEC) nanoparticles [65]. The same energy per unit volume is required to obtain an identical result independent of the scale of processing when ultrasound was applied to the formulation at the same parameter configuration. This will allow for a linear scaleup of the optimized parameter configuration to the full commercial scale [66, 67]. The bulk production of CNP can be produced by switching from the conventional ultrasonic horn (CH) to the half-wave Barbell Horn (HBH) [68]. This can increase the processing capacity by a factor of about 2(D_hbh_/D_ch_)^2^, where D_ch_ and D_hbh_ are output tip diameters of the two horns. A scale-up factor of 50–60 can be achieved through this process, making it possible to directly transfer high amplitude ultrasonic process from laboratory to industrial production [67, 68]. The bulk production of CNP can therefore be achieved, with production increasing by a factor of about 11 from laboratory scale to bench scale and by another factor of about five from bench to industrial scale [68]. The ability to scale-up the ultrasonic horn dimensions without reducing the amplitude and product quality is essential for industrial implementation. With the low energy consumption, low cost of capital and operating cost and the use of low-cost material such as; okra (US$ 1/kg); oranges (US$ 2/kg); pineapple (US$ 0.75 and palm wine (1 cent/litre). This method can be seen as a green and cost-effective compared to conventional methods.

## Conclusions

CNF is a potential material for EOR, it can be used as an alternative to recover oil from harsh reservoir condition where the application of conventional techniques has proved difficult. From the experimental results, it can be concluded that; The zeta potential result shows that CNF is stable, and the surface charge signifies long term stability of the fluid when injected into oil field reservoirs. The viscosity increased with increase in concentration and decreased with an increase in the shear rate for all the solutions, but the viscosity of CNF was higher compared to okra and xanthan. The results signify that temperature, CNF, and electrolyte concentration were the main factors that influenced the IFT reduction. As the IFT decreased with increase in the concentration of CNF, electrolyte, and temperature. The pressure drop data shows the stability of CNF at 120°C and the formation of oil bank was enough to increase the oil recovery by 20%. CNF was found to be very effective in mobilizing residual oil at HTHP reservoir condition. It can, therefore, be concluded from this experiment work that, the method applied herein is easier, cost-effective, can reduce energy consumption making the method economically advantageous compared to conventional methods.

## Supporting information

S1 File (XLSX)
